# Classification and Prediction of Brain Disorders Using Functional Connectivity: Promising but Challenging

**DOI:** 10.3389/fnins.2018.00525

**Published:** 2018-08-06

**Authors:** Yuhui Du, Zening Fu, Vince D. Calhoun

**Affiliations:** ^1^The Mind Research Network, Albuquerque, NM, United States; ^2^School of Computer & Information Technology, Shanxi University, Taiyuan, China; ^3^Department of Electrical and Computer Engineering, University of New Mexico, Albuquerque, NM, United States

**Keywords:** fMRI, functional connectivity, biomarker, classification, brain disorders

## Abstract

Brain functional imaging data, especially functional magnetic resonance imaging (fMRI) data, have been employed to reflect functional integration of the brain. Alteration in brain functional connectivity (FC) is expected to provide potential biomarkers for classifying or predicting brain disorders. In this paper, we present a comprehensive review in order to provide guidance about the available brain FC measures and typical classification strategies. We survey the state-of-the-art FC analysis methods including widely used static functional connectivity (SFC) and more recently proposed dynamic functional connectivity (DFC). Temporal correlations among regions of interest (ROIs), data-driven spatial network and functional network connectivity (FNC) are often computed to reflect SFC from different angles. SFC can be extended to DFC using a sliding-window framework, and intrinsic connectivity states along the time-varying connectivity patterns are typically extracted using clustering or decomposition approaches. We also briefly summarize window-less DFC approaches. Subsequently, we highlight various strategies for feature selection including the filter, wrapper and embedded methods. In terms of model building, we include traditional classifiers as well as more recently applied deep learning methods. Moreover, we review representative applications with remarkable classification accuracy for psychosis and mood disorders, neurodevelopmental disorder, and neurological disorders using fMRI data. Schizophrenia, bipolar disorder, autism spectrum disorder (ASD), attention deficit hyperactivity disorder (ADHD), Alzheimer's disease and mild cognitive impairment (MCI) are discussed. Finally, challenges in the field are pointed out with respect to the inaccurate diagnosis labeling, the abundant number of possible features and the difficulty in validation. Some suggestions for future work are also provided.

## Introduction

Brain disorders such as schizophrenia (SZ) and bipolar disorder (BP) are considered in terms of disruptions of the normal-range operation of brain functions. While psychiatric disorders are diagnosed based on symptom scores from clinical interview, there are no existing gold standards that can be used for definitive validation. Brain functional neuroimaging techniques including functional magnetic resonance imaging (fMRI) (Lee et al., 2013; Power et al., 2014b), positron emission tomography (PET), and electroencephalography (EEG) have become important tools in investigating brain disease (Abi-Dargham and Horga, 2016). There is much hope that brain functional connectivity revealed using functional neuroimaging data can be used to characterize brain function abnormality and in turn benefit diagnosis and treatment (Deco and Kringelbach, 2014). Among diverse modalities, fMRI enables non-invasive investigation of brain function with high spatial resolution and has been widely used to detect and characterize brain networks or connectivity among functionally interconnected regions. Investigating differences in functional network (or connectivity) between disorders such as SZ and BP may provide new insights into their disease mechanisms (Birur et al., 2017). Furthermore, the identified changes in connectivity measures may be useful as biomarkers which can be employed to classify individual patients using machine learning methods (Arbabshirani et al., 2017; Stephan et al., 2017). In this paper, we restrict our review to fMRI data, but some methods are able to be easily expanded to other brain functional imaging modalities as well.

There have been a variety of methods proposed to measure functional connectivity (FC) among brain regions using fMRI data (Van Den Heuvel and Hulshoff Pol, 2010; Smith et al., 2013; Calhoun and De Lacy, 2017). While different approaches have different assumptions and advantages, a detailed review is important to help us understand the ways in which these approaches have been used. How to select features from a large amount of measures as biomarker for building model to classify or predict brain disorders is an important and challenging problem. Classification and prediction are two forms of analysis which are used for building models to separate classes and to predict future outcomes. Generally, classification is to classify categorical disease labels that have been already acquired concurrently with or prior to the scan, while prediction is to predict unknown disease labels, future progression, or continuous-valued functions. Compared with classification, prediction is harder but more promising for clinical utility. In the context of neuroimaging, although increasing studies have tended to shift their concentration to the prediction problem, the majority of previous studies on brain disorders focused on identifying neuromarkers for classifying different groups. In this paper, we primarily aim to present a comprehensive review summarizing various brain functional connectivity measures and typical classification strategies, in order to provide guidance in this field. It is worth noting that most of the measures and strategies used in the classification problem can also be applied or extended to the prediction problem. We also survey recent exciting applications that employed fMRI data to differentiate mental disorders and other brain diseases. The challenges and difficulties as well as potential solutions are pointed out in the end.

## Functional connectivity measures from fMRI data

**Figure 1:**
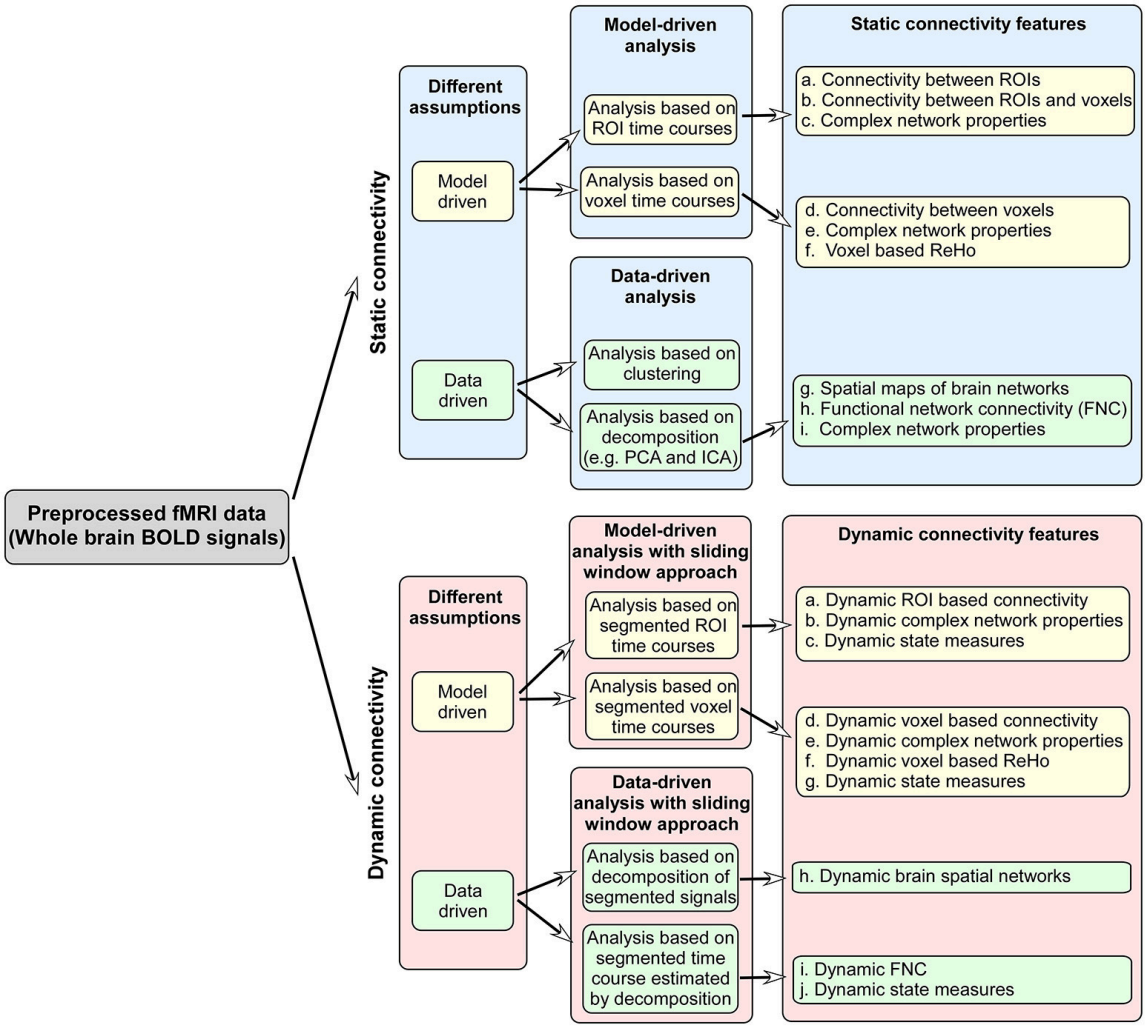
The primary functional connectivity analysis methods and possible connectivity features used for classification/prediction problem.

Functional connectivity reflects the organization and inter-relationship of spatially separated brain regions. Methods for measuring and delineating functional connectivity play a key role, since the used measures may greatly affect the identification of biomarkers and the accuracy of individual-subject classification and prediction. Typically, functional connectivity is assumed to be stationary over the scanning time (usually several minutes), and most previous fMRI studies applied a static functional connectivity (SFC) analysis. Until recently, more emerging exciting work have proven that regarding brain functional connectivity as dynamic over time can be successful in uncovering the disruptions to the normal human brain in disease condition (Calhoun et al., 2014). Figure 1 summarizes the primary functional connectivity analysis methods and possible connectivity features used for classification/prediction problem.

### Static functional connectivity analyses

From a view of methodology, there are generally three kinds of strategies analyzing SFC (Calhoun and De Lacy, 2017). The first is a model-driven strategy which uses prior knowledge to decide sets of brain regions/voxels and then limit connectivity analysis to some specific regions/voxels. The second approach is more data-driven and maps whole brain functional networks using decomposition or clustering methods. In such case, brain voxels assigned to the same component or cluster reflect regions which are highly correlated. The third combines the idea of the above two strategies, which firstly extracts co-activated regions using a data-driven method and then estimates functional connectivity among the regions. We outline several typical methods as below.

#### Model-driven analysis for assessing connectivity among regions or seeds

Brain functional connectivity analysis among *a priori* regions of interest (ROIs) or voxels (Poldrack, 2007) is the most widely applied model-driven method. Three key steps include the determination of locations and shapes of ROIs or the locations of voxels, the computation of representative time series of ROIs or voxels, and the assessment of connectivity (or coupling) among different ROIs or connectivity between each seed (ROI or voxel) and all other voxels within brain. As such, the resulting functional connectivity strengths reflect the temporal fluctuation relations among the selected voxels or regions. ROI-based functional connectivity strengths can be easily taken as features in classification and prediction problems, since the corresponding connectivity features of a new/testing subject can be directly computed between the brain regions (or voxels) selected using the training subjects. While ROIs and voxels are usually determined by subjective experience and prior knowledge, the resulting functional connectivity can be greatly sensitive to the empirical selection and show a very different pattern for small changes in the ROIs. Hence, how to decide a reasonable region including voxels with consistent brain function is a challenge. Considerable research work (Thirion et al., 2014; Glasser et al., 2016) has attempted to delineate a parcellation of brain by employing information of multiple modalities of imaging, however, inconsistencies still exists. The representative time series of voxels in one region can be calculated as the mean of all voxels' time series or the first principal component of all voxels' time series using principal component analysis (PCA). Although averaging and PCA can decrease the noise effect in the representative time series of ROIs to some extent, the obtained functional connectivity can still be related to noise. Functional connectivity between two representative time series is mostly estimated by computing correlations to measure their linear relationship, but also can be assessed by mutual information to identify non-linear relationships (Wang et al., 2014). Coherence estimates the linear relationship in the frequency domain (Sun et al., 2004), and connectivity within a specific frequency can be achieved by methods such as wavelet decomposition (Skidmore et al., 2011). It is worth noting that different measurements may reflect disparate connectivity meaning. In addition to the above computation steps, different preprocessing strategies also could affect the resulting functional connectivity strengths. Whether regressing out global mean is a controversial issue (Murphy et al., 2009; Hayasaka, 2013) and how to remove out head motion also deserve further investigation (Friston et al., 1996; Power et al., 2014a). These shortcomings should be carefully addressed while conducting analyses using the method.

#### Data-driven analysis for estimating spatial functional network maps

In contrast to model-driven methods, data-driven approaches estimating functional networks do not require the specification of predefined brain regions or voxels. These popular approaches include spatial independent component analysis (ICA) (Calhoun et al., 2001; Calhoun and Adali, 2012; Du and Fan, 2013; Du et al., 2016; Calhoun and De Lacy, 2017), principle component analysis (PCA), and clustering methods (Van Den Heuvel et al., 2008; Du et al., 2014c). In particular, ICA is a widely used approach that has shown great promise in identifying network-based biomarkers of psychiatric disorders such as schizophrenia (SZ) (Garrity et al., 2007; Ongür et al., 2010; Calhoun et al., 2011; Khadka et al., 2013; Meda et al., 2014; Du et al., 2015b, 2018). Spatial ICA of an individual-subject's fMRI data decomposes the fMRI data matrix (time points × voxels) into a linear combination of multiple maximally spatially independent components (ICs), of which meaningful ICs can be regarded as brain functional networks. In each network, the voxels with greater Z-scores tend to have higher intra-connectivity (or co-activation) (Du et al., 2018) and can be interpreted as a weighted seed maps (Joel et al., 2011). The mixing matrix in the decomposition includes the time series of the ICs, where each time series reflects the temporal fluctuation of each IC. In addition to less prior knowledge needed in advance, other advantages of ICA relative to the ROI-based method include (1) simultaneous estimation of multiple networks from whole-brain data, (2) overlapping components, which provide a spatial filtering of artifacts (Sochat et al., 2014; Du Y. H. et al., 2016a) or potentially interesting overlapping networks (Xu et al., 2013), and (3) adaptivity of components among subjects, allowing for inter-subject variability in regions to be captured (Allen et al., 2012).

The primary shortcoming of applying ICA on fMRI data is that ICA generates ICs in an arbitrary order. To solve the problem, two strategies are typically adopted in fMRI studies with multiple subjects (Calhoun et al., 2009b) to make ICs of different subjects comparable. The first strategy is to perform ICA for each subject separately, and then establish correspondence of ICs across subjects using methods such as subjective identification (McKeown et al., 1998; Calhoun, 2001), clustering (Moritz et al., 2003; Esposito et al., 2005; De Martino et al., 2007), and automated matching based on reproducibility (Yang et al., 2008). These methods could be sensitive to different source separations in multiple ICA decompositions of different subjects. For instance, one single IC detected for a certain subject may be split into several ICs including smaller active areas with closely related time courses for other subjects (McKeown et al., 1998), making it difficult if not impossible to establish correspondence among ICs of different subjects. The second strategy, often referred to as group ICA, implements one ICA on all subjects' data and then obtains subject-specific ICs from the group-level ICs somehow, which establishes direct correspondence of ICs across different subjects. The fMRI data of multiple subjects are typically grouped in three different ways with distinct hypotheses imposed upon multi-subject fMRI data, including spatial concatenation, temporal concatenation, and tensor organization. The spatial concatenation method concatenates multi-subject fMRI data along the spatial dimension supposing that corresponding ICs of all subjects have common temporal information (Svensén et al., 2002). The more frequently applied temporal concatenation method concatenates the multi-subject fMRI data along the temporal dimension (Calhoun et al., 2001, 2009b; Beckmann et al., 2009), followed by estimation of single-subject maps and time courses using an approach called back-reconstruction which includes PCA-based methods (Calhoun et al., 2001), spatio-temporal (dual)-regression (Beckmann et al., 2009; Erhardt et al., 2011) and group information guided ICA (GIG-ICA) (Du and Fan, 2013; Du et al., 2014a, 2015b; Du Y. H. et al., 2016a). Each of these can be considered as providing a different balance between ensuring matches via a group model and allowing individual subject variability to be captured. GIG-ICA is one of the more flexible approaches and estimates the subject-specific ICs by optimizing the independence measure of multiple ICs for each subject while preserving the correspondence of ICs across different subjects. GIG-ICA has been shown to well represent individual subject maps and provides an improved approach for addressing individual subject artifacts than single-subject ICA followed by group ICA (Du and Fan, 2013; Du Y. H. et al., 2016a). The tensor probabilistic ICA method stacks the original multi-subject fMRI data along a separate third dimension with a hypothesis that different subjects have common group spatial ICs and time courses but subject specific loading parameters (Beckmann and Smith, 2005; Lee et al., 2008).

Independent vector analysis (IVA) is another method which optimizes the independence among each subject's components and the dependence among corresponding components of different subjects. Several advancements of IVA have been made for achieving reliable source separation for linearly dependent Gaussian and non-Gaussian sources (Anderson et al., 2010; Dea et al., 2011; Li et al., 2011; Adali et al., 2014; Anderson M. et al., 2014; Boukouvalas et al., 2015). Among those, IVA-GL, which is a combination of two IVA algorithms, IVA with multivariate Gaussian component vectors (IVA-G) (Anderson et al., 2012) and IVA with multivariate Laplace component vectors (IVA-L) (Lee et al., 2008), provides an attractive tradeoff in terms of complexity and performance. A direct comparison of IVA and GIG-ICA was performed in recent work (Du et al., 2017b) which emphasized the advantages of the two approaches. For sources with slight or moderate inter-subject spatial variability, GIG-ICA obtained components with higher accuracy than IVA. For datasets where all subjects had a subject-unique source with large inter-subject spatial variability, IVA showed better performance in the component/time courses (TC) accuracy of the unique source, although GIG-ICA in general still performed better for other subject-common sources compared to IVA. Therefore, a framework that leverages the strengths of IVA and GIG-ICA is expected to achieve high accuracy for both subject-common and subject-unique networks.

It is also well-acknowledged that another pitfall of data-driven approaches (Calhoun and De Lacy, 2017) is the requirement to select a certain model order (e.g., the number of components in decomposition methods or the number of clusters in clustering methods) that may greatly affect the resulting brain network maps. While employing ICA to extract functional networks, the number of components is typically estimated using information-theoretic principles, such as a modified minimum description length (MDL) criteria (Li et al., 2007). Since different estimation methods result in different numbers of components (Zuo et al., 2010), it is important to consider the impact of the model order. Moreover, it is likely that a single model order is not the best solution, rather one can consider evaluating the impact of a range of model orders which enables a hierarchical evaluation of the brain's spatial organization (Ma et al., 2011; Calhoun and De Lacy, 2017).

It is known that features are required to be comparable across subjects for the purpose of classification or prediction. In decomposition-based methods, how to propagate components (indicating functional networks) to a new subject that is not included in the original training set is an important issue. In the case of applying individual ICA on each subject separately, the obtained components from each coming subject have to be well-matched with the components from the training set using some matching rules so that their used features are consistent. In group ICA framework, there are several ways to do this, one can use spatio-temporal regression to generate spatial and temporal features from new subjects (Erhardt et al., 2011). Another approach is to use spatially constrained ICA (Lin et al., 2010; Du and Fan, 2013; Du et al., 2014b, 2015b; Du Y. H. et al., 2016a). The latter approach is more optimal as individual data sets will have results that are optimized for independence, and will also provide spatial and temporal features that are adapted to each individual subject. A classification study using this framework can be found in Du et al. (2015b).

#### Functional network connectivity analysis

Functional network connectivity (FNC) (Jafri et al., 2008; Allen et al., 2011) analysis employs a strategy that combines model-driven and data-driven methods. The framework typically includes two steps. It first performs group ICA on fMRI data of multiple subjects, resulting in subject-specific functional networks (indicated by ICs) and their associated fluctuations (reflected by TCs). Then, the connectivity between any two networks can be obtained by computing connectivity measure such as Pearson correlation between their post-processed TCs, resulting in a connectivity matrix including connectivity strengths among all networks. Similar to the ROI-based method, FNC also reflects temporal connectivity among different brain regions. The difference between the ROI-based and FNC method is that a data-driven method is applied to fMRI data in the FNC analysis to generate brain regions that are functionally co-activated (i.e., regions in one network), while in ROI-based method brain regions are usually decided via prior knowledge (e.g., brain atlas) rather than using the in-house fMRI data. Similar to ICA, it is necessary to determine the number of components in advance in the FNC method. FNC approaches typically use, a high model order (e.g., 100 or larger) to provide a more detailed parcellation of the brain.

#### Other functional connectivity measures

In addition to the typical approaches for assessing functional connectivity (e.g. correlation) other meaningful measurements have also been proposed. For example, the regional homogeneity (ReHo) (Zang et al., 2004) has been proposed to reflect regional functional connectivity (or co-activation) where Kendall's coefficient concordance (KCC) is used to measure the similarity of the time series of a given voxel to those of its nearest neighbors. A similar approach is Cohe-ReHo (Liu et al., 2010) computed based on coherence metrics. Regional connectivity may serve as features for differentiating patients and healthy controls. Moreover, after functional connectivity matrices are obtained from either model-driven or data-driven techniques, graph-theory derived metrics (Liu et al., 2008; Lynall et al., 2010; Yu Q. et al., 2013) such as the averaged node strength, clustering coefficient, global efficiency, and local efficiency (Rubinov and Sporns, 2010) can be calculated. These graph-based measures provide powerful features which integrate across the whole brain and can be used in classifying and predicting individual patients.

### Dynamic (time-resolved) functional connectivity analyses

All of the above mentioned analysis approaches estimate brain functional connectivity by computing an average of the full time series (e.g., computing Pearson correlation between two ROIs using BOLD signals within 5 or 10 min) and generate a static value to reflect the connection strength. In recent years, there have been much interests in computing time-resolved connectivity measures and successful applications in identifying biomarkers from dynamic connectivity (Chang and Glover, 2010; Sakoglu et al., 2010; Allen et al., 2014; Zalesky et al., 2014; Du et al., 2015a; Sadaghiani et al., 2015; Du Y. H. et al., 2016b). In such analysis, brain functional connectivity can vary within a short period (e.g., tens of seconds) rather than be considered as static over time. Such results tend to further expand the available information, and avoid the strong assumption that brain activity is static over time.

While dynamic functional connectivity (DFC) has emerged as a promising topic in the recent fMRI literature, there are also some critical comments on the theory of dynamic connectivity. Laumann et al. (2017) suggested that correlations measured by resting-state BOLD are relatively stable over short timescales and may not reflect moment-to-moment changes in cognitive content. Though this issue is still not completely settled, many new studies have shown a relationship between behavior, emotion, and cognition during rest with dynamic connectivity features, giving us confidence in its potential utility. In addition, since dynamic connectivity has shown to be a useful tool for identifying biomarkers, we introduce some typical approaches and applications in terms of dynamic connectivity.

#### Sliding time-window based dynamic connectivity analysis

There are numerous methods which can be used to estimate DFC (Calhoun et al., 2014; Chen J. E. et al., 2017; Preti et al., 2017). The sliding time-window technique (Sakoglu et al., 2010; Hutchison et al., 2013; Hindriks et al., 2016; Shakil et al., 2016) is the most widely used. By assessing functional connectivity in different time-windows, one can easily expand existing static connectivity strategies to be time-resolved. DFC can then be evaluated by measuring functional connectivity among ROIs or voxels in a sliding window yielding multiple connectivity matrices (Du Y. H. et al., 2016b; Du et al., 2017a,c), performing ICA (or IVA) on fMRI data in different windows to generate dynamic spatial network patterns (Kiviniemi et al., 2011), or segmenting time series of networks (i.e., ICs) into short time series and then computing time-varying FNC (Allen et al., 2014). The sliding-window technique has also been applied to evaluate ReHo and brain graph, yielding time-varying ReHo values (Deng et al., 2016) and time-varying graphs (Yu Q. B. et al., 2015; Du Y. H. et al., 2016b).

Dynamic connectivity analyses among brain regions and networks have attracted increasing interests. Various approaches to further investigate the time-varying connectivity patterns is a topic of ongoing work. Different connectivity states, reflecting specific configurations of connected regions, can be revealed by *post-hoc* analyses of dynamic connectivity (Calhoun et al., 2014; Damaraju et al., 2014; Rashid et al., 2014; Du et al., 2015a, 2017c; Yu Q. B. et al., 2015; Du Y. H. et al., 2016b). Therefore, changes in connectivity states among different clinical populations might provide unique or additional biomarkers of disorders not detectable with SFC measures. Researchers have applied clustering (Allen et al., 2014; Du Y. H. et al., 2016b), principal components analysis (PCA) (Leonardi et al., 2013), Fisher discrimination dictionary learning (FDDL) (Li et al., 2014), and spatial and temporal independent components analysis (ICA) (Yaesoubi et al., 2015b; Miller et al., 2016) to extract connectivity states. These methods typically estimate connectivity states with discrepant patterns due to their different assumptions (Calhoun et al., 2014). Clustering approaches may fail to converge when working on “noisy” data that do not necessarily have desirable distributions. A more serious shortcoming of clustering is that the method always can yield a partition with any given number of clusters, regardless if the used features show patterns indicating clusters. The above mentioned decomposition-based work (Leonardi et al., 2013; Li et al., 2014; Yaesoubi et al., 2015b; Miller et al., 2016) focuses on group-level connectivity states that are common across subjects. One can also use GIG-ICA to estimate connectivity states at both group-level and subject-level (Du et al., 2017a,c). The method first computes the group-level connectivity states by analyzing multiple subjects' dynamic connectivity, and then guided by the group-level states it correspondingly estimates the subject-specific connectivity states that are independent from each other.

There has been considerable work using DFC analyses to investigate impairments in schizophrenia-spectrum and mood disorders (Damaraju et al., 2014; Rashid et al., 2014; Du Y. H. et al., 2016b; Du et al., 2017a,c) as well as classifying individual patients based on DFC measures (Rashid et al., 2016). Damaraju et al. (2014) computed dynamic FNC matrices of healthy controls (HCs) and SZ patients, and then clustered the time-varying FNC into different states, suggesting that states exhibiting cortical-subcortical negative connectivity and strong positive connectivity between sensory networks are those that show the group differences of thalamic hyperconnectivity and sensory hypoconnectivity. Rashid et al. (2014) also analyzed dynamic DFC of SZ patients and BP patients using a clustering method, and found that SZ patients showed more changes than BP subjects, including both hyper and hypo connectivity in one common connectivity state. Du Y. H. et al. (2016b) estimated dynamic connectivity within the default mode network (DMN) of 82 HCs and 82 SZ patients using a ROI-based method, and then applied K-means to extract connectivity states. The results showed that HCs spent more time in a state that reflected stronger connectivity between anterior and posterior brain regions, while SZ patients spent more time in a disconnected state. Another study (Du et al., 2017c) extracted connectivity states from whole-brain ROI-based DFC of 238 HCs, 140 bipolar disorder with psychosis (BPP), 132 schizoaffective disorder (SAD) and 113 SZ patients using GIG-ICA. Results showed that DFC provided more informative measures than the SFC method. Diagnosis-related connectivity states were evident using DFC analysis. For the dominant state consistent across groups, 22 instances of hypoconnectivity (with decreasing trends from HC to BPP to SAD to SZ) mainly involving post-central, frontal and cerebellar cortices as well as 34 examples of hyperconnectivity (with increasing trends from HC to BPP to SAD to SZ) primarily involving thalamus and temporal cortices were found. Interestingly, hypoconnectivities/hyperconnectivities also showed negative/positive correlations, respectively, with clinical symptom scores. Regarding frontal connectivities, BPP resembled HC while SAD and SZ were more similar. Using a similar framework, whole-brain DFC from resting-state fMRI data of 70 HCs, 53 individuals at clinical high-risk (CHR) for psychosis, and 58 early illness schizophrenia (ESZ) patients were utilized to estimate the inherent connectivity states, and then group differences were identified (Du et al., 2017a). The work found widespread connectivity alterations in both CHR and ESZ groups, and ESZ patients generally showed more connectivity differences with larger changes than CHR individuals relative to controls. Inspired by these studies, we believe that changes of connections within states, temporal measures such as dwell time in different states, as well as disease-specific states in dynamic connectivity analysis are able to provide interesting features for classification of diseases in future.

Furthermore, the time-varying patterns in brain activity and their relationships with time-varying brain connectivity are also important for advancing our understanding of brain networks and the underlying mechanism of brain dynamics. A recent study (Fu et al., 2017) developed a framework based on the sliding window approach for characterizing time-varying brain activity and exploring its associations with time-varying brain connectivity. This framework was applied to a resting-state fMRI dataset including 151 SZ patients and 163 age- and gender-matched HCs, suggesting that amplitude of low frequency fluctuation (ALFF) and FNC were correlated along time and these relationships are significantly changed in SZ.

#### Windowless methods for extracting dynamic connectivity

The above mentioned sliding time-window methods have been extensively used and are successful to estimate dynamic connectivity. However, there is an apparent limit in lacking standards for setting the window length, although previous studies have suggested 30–60 s of window length that are feasible in capturing DFC (Zalesky and Breakspear, 2015). If the window length is too short, the time points in each window could be too few to generate robust estimation of connectivity strengths. In contrast, long window length might decrease the temporal variations of functional connectivity, consequently hindering from detecting effective connectivity states.

Several windowless-based methods have been proposed to avoid the problem in selecting the window length. The recently proposed time-frequency analysis (Yaesoubi et al., 2015a) explored the connectivity by using multiple frequencies, which can be conceptually seen as adapting the observation window to the frequency content of the original time courses. Bayesian approach (Robinson et al., 2015; Taghia et al., 2017) has also been employed to study dynamic connectivity, which regards extracting time-varying functional networks as selecting dynamic models in the Bayesian setting. More recently, a new approach (Yaesoubi et al., 2018) was proposed to estimate DFC with the main advantage of capturing connectivity with arbitrary rates of change. In the approaches based on windowing operation, observable rate of change is driven by the length of the window, but in this approach there is no requirement for a windowing operation.

## Classification or prediction strategies

Brain disorders cause serious impairments or debilitating behavior and represent a major health and financial burden globally (Vigo et al., 2016). In the United States, brain disorders (such as the symptoms, diagnosis, and treatments) are typically defined using the Diagnostic and Statistical Manual (DSM) (American Psychiatric Association, 2013). There are also some alternatives offer standard criteria for the classification of brain disorders, such as ICD-10 Classification of Mental and Behavioral Disorders, produced by the world health organization (WHO). However, over the years new knowledge is continuously added, resulting in changes in the diagnosis and disease classification (e.g., some are not valid, some are changed, and new ones appear). In addition, many mental illnesses are diagnosed based largely on symptoms, rather than biological criteria. More recently, there has been a focus on the importance of looking across disorders and also on continuous measures of assessment in both health and disease, e.g., the research domain criterion (RDoC) (Insel and Cuthbert, 2015). In this context, there has been an increasing trend to identify biological markers. Brain functional connectivity has been of great interest in the search for markers of numerous brain disorders. In the following, we will review some commonly used feature selection and classification (or prediction) strategies in fMRI functional connectivity based brain disorder studies. Several key aspects of feature selection methods and classifiers are compared and their promise and pitfall are discussed.

### Feature selection strategies

The properties of fMRI data make feature selection especially important in the classification and prediction (Van Schooten et al., 2014). The dimension of functional connectivity is large even if ones only evaluate connectivity between defined ROIs. If the functional connectivity is calculated between voxels, the number of features will go up (potentially millions of features). Functional connectivity relating to a specific brain disorder often focuses on a small portion of all possible connections/associations. In that case, if all functional connections are used as features in a classifier, it would cause an overfitting problem since algorithm tries to fit the classifier to every feature even the irrelevant ones. If the classifier variables are overfitted to the training samples, they might work poorly on the samples not in the training sets, resulting in unsatisfied performance in classification. Another problem is that functional connectivity might provide substantial redundant information for classification. Using all connections as features with redundant information might be detrimental to the results of classification. Considering this, it is important to incorporate good feature selection strategies to identify appropriate functional connectivity features for the classification of brain disorders. Table 1 summarize the properties of different feature selection methods.

**Table 1:** Summary of the feature selection methods.

**Feature selection**	**Popular methods**	**Relationship with classifier**	**Computational complexity**	**Models feature dependencies**	**Overfitting problem**	**Other pros and cons**
Filter methods	•Statistical test •Fisher score •Correlation coefficient	Independent	Lowest	No	Least likely	Pros: scalable Cons: features might not be optimal
Wrapper methods	•RFE •Sequential feature selection •Genetic algorithm	Dependent	Highest	Yes	Most likely	Pros: simple and less prone to local optima
Embedded methods	•LASSO regularization •Decision tree	Dependent	Middle	Yes	Likely	Cons: complexity than wrapper methods, specific to a learning model

#### Filter methods

A widely applied feature selection strategy is filter-based method, where feature selection is independent from classifier/model building (Guyon and Elisseeff, 2003). They use the general characteristics of dataset and assign proxy measures to features from which a number of features with top scores are selected. A good filter method is sensitive to the discretionary power so as to suppress the least interesting features. The most popular filter method is to use group-level statistical tests. Generally, functional connectivity with group difference are first identified using different statistical tests such as *t*-test, Welch's *t*-test and ranksum-test and then these functional connections are used as input features of classification approaches (Calhoun et al., 2008; Anderson et al., 2011; Bassett et al., 2012; Du et al., 2012; Arbabshirani et al., 2013; Fekete et al., 2013; Guo H. et al., 2014; Dyrba et al., 2015). A major problem with this strategy is that group difference is sometimes investigated using whole data (Arbabshirani et al., 2017). That is, the label information for testing samples is used for feature selection, which will result in biased classification results. Another issue is that features are often selected based on their *p*-values. However, functional connectivities which show small *p*-values for group comparisons do not necessarily reflect those with the largest discrimination power. One previous study in our group has shown that features can have different distributions but comparable group means for different cohorts (Arbabshirani et al., 2017). This type of features might have a large *p*-value of statistical tests but good classification performance. There are also other filter methods used in the classification of brain disorders. Fisher score is a univariate feature selection algorithm which has been applied to determine the discriminatory power of features between two groups with equal probability (Gu et al., 2012; Khazaee et al., 2015). Correlation-based feature selection (CFS) is a simple algorithm which ranks features based on a hypothesis that good feature subsets contain features highly correlated with the classification (Hall, 1999; Shen et al., 2010; Tang et al., 2012; Su et al., 2013; Challis et al., 2015). RELIEF based algorithms are another large family of filter methods which estimate the scores of features according to how well their values distinguish between instances (Kira and Rendell, 1992). These methods are not dependent on heuristics, run in low-order polynomial time, and are noise-tolerant and robust to feature interactions, as well as being applicable for binary or continuous data (Kira and Rendell, 1992). The minimum redundancy, maximum relevance (mRMR) algorithm has also been used for the feature selection (Lord et al., 2012). This method uses each feature's predictive power and the mutual information between features to rank the most relevant features. mRMR can achieve satisfactory results compared with an exhaustive search, without the increase in time cost for ordering the feature list. The major advantages of filter methods are their effectiveness in computation time and their robustness to overfitting (Hamon, 2013). However, filter methods also have several drawbacks. First, the features selected by filter methods are not optimized to suit any specific classifier. Secondly, some of the filter methods tend to select redundant features since they ignore the relationships between features.

#### Wrapper methods and embedded methods

Wrapper methods, which involve optimizing classifiers as part of the feature selection, have also been used in the classification (Guyon and Elisseeff, 2003; Fan et al., 2011; Venkataraman et al., 2012; Yu Y. et al., 2013b). Generally, wrapper methods use classifiers or predictive model to rank features. This class of methods evaluates the classification performance of different combinations of features and tries to identify the optimal subset of features that can provide the largest discriminatory power. Since the number of possible feature combinations grows exponentially as the number of features increase, customizable heuristics and termination-conditions are typically employed in wrapper methods to avoid that the selection of features is beyond a computer's processing power. Various wrapper methods have been employed in the brain disorders classification studies. Recursive feature elimination (RFE) is the most popular used wrapper method which selects features by recursively considering smaller and smaller combinations of features (Castro et al., 2011, 2014; Ladha and Deepa, 2011; Colby et al., 2012; Dai D. et al., 2012; Du et al., 2015b). This algorithm trains classifiers using the initial set of features and ranks the features according to their importance. The least important features are then discarded and the procedure is recursively repeated using the remaining features until a pre-desired number of features is select. Another widely used wrapper method is the genetic algorithm (GA) family, which uses binary encoding and specific mutation for feature selections (Yang and Honavar, 1998). Initially, binary encoded subsets of predictors (a feature is either included or not in the subset) are created and their corresponding fitness values, such as classification accuracy, are calculated. The encoded subsets then undergo cross-over and are subject to random mutations. This process is repeated again and again to create better subsets of predictors. Wrapper methods tend to select better performing features than filter methods and can provide the best feature selections specific for a particular type of classifier. However, wrapper methods also have two major shortcomings. First, wrapper methods might overfit if the number of observations is not large. And secondly, wrapper methods are computationally much more expensive since they need to create classifiers recursively.

Embedded methods, which combine classification and feature selection into the decision process, have also been applied to classification (Lal et al., 2006). Embedded methods are similar to wrapper methods since both of them incorporate feature selection into the classifier construction process. However, wrapper methods use a learning machine to measure the quality of subsets of features without incorporating knowledge about the specific structure of the classification or regression function; therefore they can combine with any learning machine. In embedded methods, the learning part and the feature selection part cannot be separated. An intrinsic model building metric is used during the learning process for embedded methods in which the feature selections are specific to given learning machines. A common category of embedded methods is using a regularization penalty to enforce the sparsity of features in order to identify features with more discriminatory power. The most popular embedded method with regularization penalty is the least absolute shrinkage and selection operator (LASSO) method (Tibshirani, 1996; Jie et al., 2014; Watanabe et al., 2014; Rosa et al., 2015; Fonti and Belitser, 2017). The LASSO method builds a linear model and penalizes the regression weights using L1 penalty. Amount of weights are shrunk to zero and those features with non-zero weights are selected finally. Ridge regression is another embedded method used for the feature selection (Yu and Liu, 2003; Ng, 2004). Similar to LASSO method, ridge regression shrinks the regression weights by incorporating a penalty. However, the ridge penalty behaves differently than LASSO penalty. The ridge penalty would be more likely to select features with high correlations than the LASSO penalty and tend to provide better classification performance. The elastic net algorithm is an extension of LASSO (Zou and Hastie, 2005; Gheiratmand et al., 2017; Teipel et al., 2017). It overcomes LASSO limitations on the feature number selections and the stabilization of feature selection by using a combination of LASSO and ridge regression methods. Since embedded methods select features specific to the classifiers, they are much faster and less computationally expensive.

### Classification and prediction models

#### Traditional classifiers

A wide range of classifiers has been applied in the classification of brain disorders. Support vector machine (SVM) is so far the most popular method (Lord et al., 2012; Anderson and Cohen, 2013; Yu Y. et al., 2013b; Watanabe et al., 2014; Du et al., 2015b; Dyrba et al., 2015; Khazaee et al., 2015; Liu et al., 2015; Sacchet et al., 2015; Cabral et al., 2016). SVM is a type of supervised learning classifier with learning algorithms used for classification and regression (Cortes and Vapnik, 1995b). Standard SVM is a binary classifier which generalizes the optimally separating hyperplane to better separate different groups of data. The basic idea of SVM is to find an observation of one class which is closest to an observation from the other class. The hyperplane is drawn in a way that maximizes the distance between these observations so that the hyperplane can separate the observations into different sides. Since a “slack variable” is used in the SVM classifier, SVM allows overlaps between different groups. There is no assumption needed for the SVM classifier, making it a very flexible method. However, it is also hard to interpret the results from SVM compared with the other traditional classifiers. The original SVM classifier is a linear classifier. By incorporating the different kernel functions to maximum-margin hyperplanes, SVM can become non-linear classifiers. The kernel functions transfer the original features space to a higher-dimensional feature space so that the algorithm can fit the maximum-margin hyperplane in a new feature space. Several common kernels are widely used in SVM, such as polynomial kernel, sigmoid kernel, and Gaussian RBF kernel. The choice of kernel is crucial for building a successful SVM-based classifier. Different types of the kernel will be suitable for different studies depending on the characteristics of features. SVM with different kernels will have different hyperparameters needed to be optimized. For example, SVM with linear kernel has only one hyperparameter to be adjusted which is called soft margin. In addition, SVM approaches using non-linear kernels have one or more additional hyperparameters to be tuned. The optimization of hyperparameters is usually based on a grid search over pre-provided candidate values. It is very important in SVM as these parameters significantly influence classification performance and accuracy.

Linear discriminant analysis (LDA) is another widely used classifier (Dai Z. et al., 2012; Cetin et al., 2016; De Marco et al., 2017; Qureshi et al., 2017a; Wang et al., 2017), which projects features into a lower-dimensional space in which different groups of data can be maximally separately (Altman et al., 1994). LDA is a generalization of Fisher's linear discriminant and is based on the concept of searching for a linear combination of features that separate two groups (Mika et al., 1999). LDA explains the group labels by the values of continuous independent variables. By projecting the data into a lower-dimensional space, LDA can avoid the overfitting problem and reduce the overall computational costs. LDA is very similar to principal component analysis (PCA). PCA is used for finding the axes that maximize the variance of data while LDA is used find finding the axes that maximize the separation between multiple groups. LDA also has two major limitations. First, LDA requires the assumption of a common covariance structure in the groups of data, which is very rare in real applications. Second, although LDA can be used for multi-class classification problem, it is more suited to the two-class problem.

#### Deep learning classifiers

Deep learning methods have attracted increasing interesting in various areas and also have been applied in the classification of brain disorders (Plis et al., 2014; Iidaka, 2015; Lecun et al., 2015; Calhoun and Sui, 2016; Hu et al., 2016; Kim et al., 2016; Han et al., 2017; Jang et al., 2017; Ju et al., 2017). In contrast to traditional machine learning methods, deep learning methods are capable of learning the optimal representation directly from the raw data through using a hierarchical structure with different levels of complexity (Lecun et al., 2015; Schmidhuber, 2015; Vieira et al., 2017). Deep learning methods apply non-linear transformations to the raw data, and the transformations provide hidden features with higher levels of abstraction, which will be with more informatics to the original input data space at the lower levels. This advantage not only helps to automatically solve difficulties in the feature selections, especially when the dimension of features is too large or when there is limited prior knowledge about the data, but also can improve classification performance compared with a traditional classifier.

The artificial neural network (ANN) is popular in the classification of patients using fMRI data (Guo H. et al., 2014; Kim et al., 2016). ANN learns to do tasks from examples by constructing layers with artificial neurons and connections between them. For example, in brain disorder classification, it learns to identify individuals with brain disorder by analyzing training subjects which are labeled as healthy or disorder and using this information to classify other individuals. An auto-encoder is a type of ANN popular used for the brain disorders classifications (Kim et al., 2016; Guo X. et al., 2017; Ju et al., 2017). This method comprises two stages. The first stage is encoding, which maps the input to a hidden representation. The second stage is decoding, which maps hidden representation back to obtain the output that is as close to the input as possible. By imposing sparsity on the hidden layers during training, an auto-encoder can learn useful structures from the input data. This allows sparse representations of inputs, which are useful in pre-training for classification tasks. Deep belief network (DBN) is another class of ANN been used in the classification of brain disorders using fMRI data (Farzi et al.), which is composed of multiple layers of latent variables and the connections between them (Hinton, 2009). A DBN is somewhat unique in that it allows undirected connections between some layers, called restricted Boltzmann machines (RBM) (Hjelm et al., 2014). DBN usually trains these layers using an unsupervised learning algorithm such as the gradient descent algorithm. Therefore, instead of using deterministic functions and the reconstruction error (like the auto-encoder), DBN is pre-trained using maximum-likelihood estimation (Vieira et al., 2017).

Several critical issues challenge the using of deep learning in classification (Schmidhuber, 2015; Vieira et al., 2017). The first challenge is the amount of time and computational resources. The number of layers, nodes and the function of each node are usually manually determined, although some automated optimization strategies have been proposed. A large number of parameters needs to be estimated in the deep learning methods, which makes them cost much more computational resources. A second challenge is the potential overfitting problem when using deep learning methods. Since the feature dimension of fMRI data is usually very large while the number of samples is relatively small, deep learning methods will tend to learn features in the data which are specific or limited to the study. Although there are several approaches developed to address this problem, such as regularization strategies and pre-selection of features (i.e., reducing the dimensionality of feature input), these approaches also introduce other critical problems, such as how to induce appropriate sparsity and how to select the best subset of features. The third challenge is the interpretability of results obtained from deep learning methods. The deep learning methods are often treated as a black box, which use consecutive non-linear transformations on the raw features to map them to another space with higher levels of abstraction. Although the model information, such as the node in the hidden layers and the connection between them, has been demonstrated to be useful for distinguishing brain disorders, it is difficult to back-construct them to the original feature space, which will result in problems of interpreting the results. Because of these issues, a deep learning method might work well in the classification of a brain disorder but does not provide any information about the underlying neuroanatomical or neurofunctional alterations. That would be of limited clinical utility (Vieira et al., 2017). Although these issues remain unsolved, deep learning methods are still with a great potential to improve the diagnosis of brain disorders and could be promising tools for advancing the knowledge of disrupted brain cognitive functions in brain disorders.

A summary of the properties of different classifier models can be found in Table 2.

**Table 2:** Summary of traditional classifiers and deep learning classifiers.

**Classifiers**	**Popular models**	**Classification performance**	**Computational complexity**	**Overfitting**	**Feature selection**	**Sensitive to redundant feature**	**Interpretability**
Traditional classifiers	•SVM •LDA •Logistic regression	Worse	Lower	Less likely	Need	More	Simple and transparency
Deep learning	•Autoencodes •DBN •CNN	Better	Higher	More likely	No need	Less	Hard and lack of transparency

### Binary classification to multi-class classification

In the context of the classification of brain disorders, the majority of the conventional studies have just focused on binary classification, in which only the comparison between patients and healthy controls was taken into account. However, from the clinical perspective, it would be more critical to identify and develop biomarkers to differentiate different brain disorders which share similar symptoms. It is also important to separate patients into different sub-groups according to the different stages of brain disorder progression. Therefore, the multi-class classification problem can be a more significant issue for real clinical utility. During the recent decade, increasing brain disorder studies have drawn their attention to multi-class classification. Since most of the traditional classifiers, such as SVM and LDA, were originally designed for binary classification problem (Cortes and Vapnik, 1995a; Mika et al., 1999), many strategies have been developed to make the traditional classifiers work for multi-class classification problems. The most commonly used strategy is to transform multi-class classification problem to binary classification problem. This strategy includes two different techniques, one-against-one and one-against-whole (Nasrabadi, 2007). The former builds binary classifiers for all pairs of groups and uses a voting scheme to make the final decision. The latter one trains a single classifier for each class (against other classes) and generate a real-value confidence score for the final decision. Although this strategy accompanied with traditional classifiers has been widely applied in numerous neuroimaging classification studies, such problem transformation is still controversial. Some other approaches have also been proposed (Hsu and Lin, 2002; Fei and Liu, 2006), but none of them have been applied to any multi-class brain disorder studies (Kumar and Gopal, 2011; Vieira et al., 2017). Compared with the traditional classifiers, deep learning classifiers are more suitable for multi-class comparison because the application of these classifiers on multi-class problems is more straightforward. In the output layer, deep learning classifiers use a softmax activation function, which can be derived by extending simple logistic regression, to represent a categorical distribution instead of group labels. In that case, the probabilities of each input feature belonging to a class are obtained from the output layer, providing a more intuitive index of multi-class membership those sophisticated indices generated from traditional classifiers (Vieira et al., 2017). Nowadays, there is a growing trend toward using deep learning classifiers to separate different brain disorders or brain disorder subtypes, or to diagnose the progression of brain disorder.

## Applications using brain functional connectivity in the classification of brain disorders

**Figure 2:**
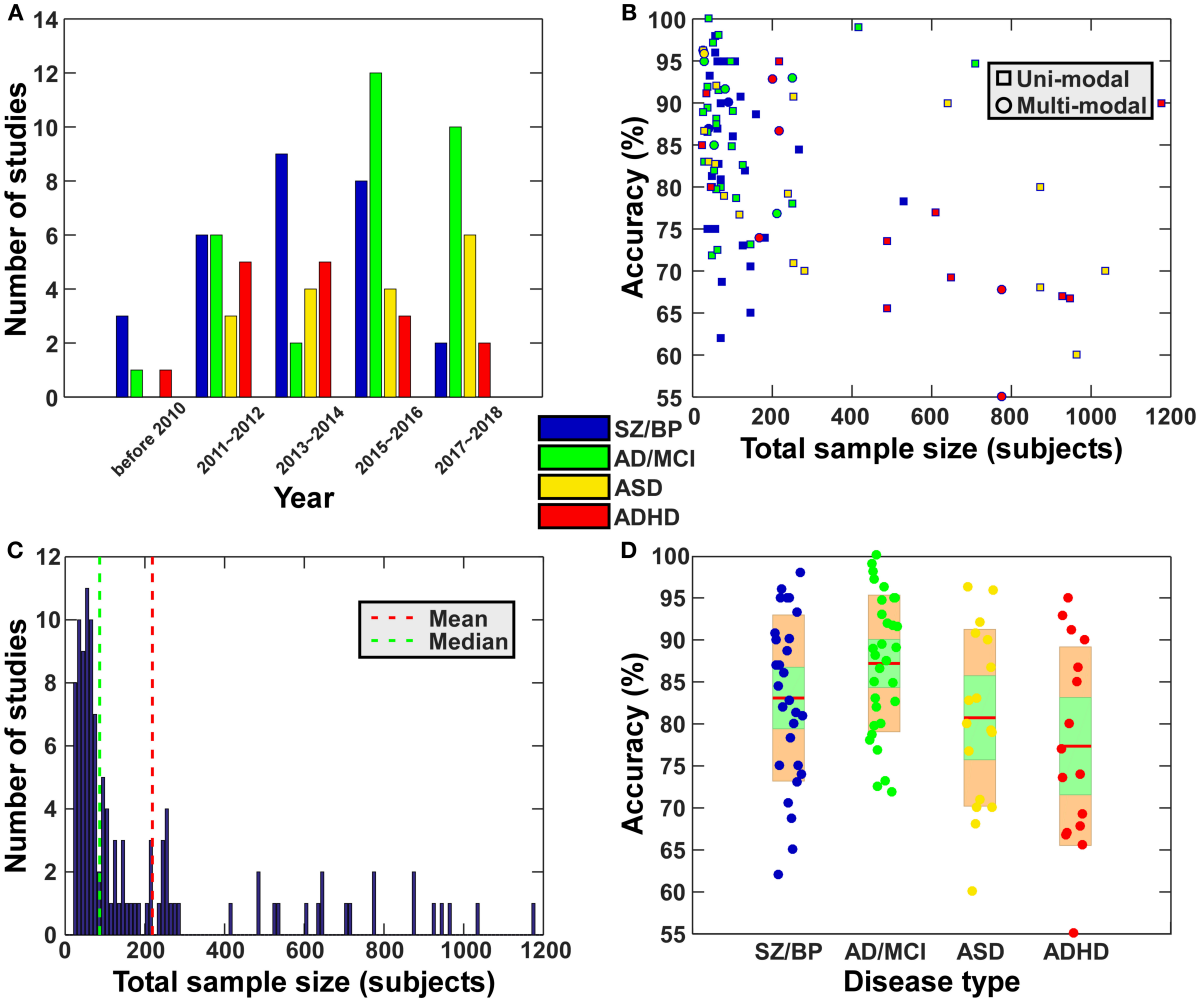
Summary of the existing application studies (included in Tables 1–6). **(A)** Total number of papers for 2-year intervals for each disease type. The legend shows the color code for each disease type. This legend also applies to subfigure **(B,D)**. **(B)** Scatter plot of the reported classification accuracy vs. the total sample size. In the subfigure **(B)**, square shape indicates study using features from one modality, while circle shape represent study using features from multiple modalities. **(C)** Histogram of the sample sizes (including all patients and healthy controls) of the surveyed studies. Vertical dashed lines indicate mean (red) and median (blue) of the sample size among all studies. **(D)** Disorder specific boxplot plots of reported classification accuracies of the surveyed papers. For each disease type, the accuracies in different studies are shown using a boxplot. Green shape means a 95% confidence interval for the mean while orange shape means standard deviation.

During the period from 1990 to 2017, more than 200 papers used functional connectivity features alone or multi-modality features including functional connectivity to classify or predict brain disorders. In this section, we primarily focus on studies working on classifying patients with a brain disorder from healthy controls (i.e., a binary classification problem), and also include some work distinguishing multiple different disorders (i.e., a multi-class classification problem). We mainly summarize studies relating to schizophrenia, bipolar disorder, autism spectrum disorder (ASD), attention deficit hyperactivity disorder (ADHD), Alzheimer's disease (AD) and mild cognitive impairment (MCI), some of which share very similar symptoms and common changes in the brain that can confound diagnosis, such as SZ vs. BP, ASD vs. ADHD, and AD vs. MCI. Although other brain disorders such as depression also deserve review in the future, our primary goal here is to provide an overview on how far brain functional connectivity features have been used to classify brain disorders and how well the classification frameworks have worked. Figure 2 and Tables 3–6 present a summary of the existing application studies that reported their classification accuracy. Regarding the performance, the average classification accuracy is around 80% for those studies, with AD/MCI related studies showing the highest accuracy. In these applications, there are trends from using connectivity features alone (e.g., spatial maps of ICA and functional connectivity) to using complex network properties (e.g., graph-theory based measures); from using static connectivity measures to using dynamic connectivity measures; from using features from single imaging modality to using features from multiple modalities; from using traditional classifiers to using more complex deep learning classifiers; and from classifying patients from healthy controls to classifying multiple groups. In each of the following subsections, we focus on some typical works in more detail to highlight these potential trends. If there are both binary and multi-class classification works, we will describe binary classification studies first. Similarly, we try to first state studies using simple features or classifiers and then that using more complex features or classifiers.

**Table 3:** Summary of functional connectivity based SZ/BP classification studies.

**References**	**Disorders**	**Features**	**Classifier**	**Sample size**	**Accuracy**
Calhoun et al. (2008)	Schizophrenia and bipolar disorder	Network maps (DMN and temporal lobe) extracted by ICA	Euclidean distance	26 HC, 21 SZ, and 14 BP	83–95%
Demirci et al. (2008)	Schizophrenia	Network maps extracted by ICA	Projection pursuit stages	36 HC and 34 SZ	80~90%
Yang et al. (2010)	Schizophrenia	Multi features include single nucleotide polymorphism, Voxels in fMRI map, and Components of fMRI activation	SVM	20 HC and 20 SZ	74~87%
Castro et al. (2011)	Schizophrenia	Activation maps extracted by GLM and networks extracted by ICA	SVM	54 HC and 52 SZ	95%
Fan et al. (2011)	Schizophrenia	Network maps extracted by ICA	SVM	31 HC and 31 SZ	87%
Bassett et al. (2012)	Schizophrenia	FC between 90 ROIs (AAL atlas)	SVM	29 HC and 29 SZ	75%
Du et al. (2012)	Schizophrenia	Network maps extracted by ICA	Fisher discriminant function	28 HC and 28 SZ	93~98%
Tang et al. (2012)	Schizophrenia	FC between 90 ROIs (AAL atlas)	SVM	22 HC and 22 SZ	93.2%
Venkataraman et al. (2012)	Schizophrenia	FC between 77 anatomical ROIs	Random Forest analysis	18 HC and 18 SZ	75%
Anderson and Cohen (2013)	Schizophrenia	Graph measures	SVM	29 HC and 19 SZ	80%
Anticevic et al. (2013)	Schizophrenia and bipolar disorder	Thalamus seed-based connectivity	SVM	90 HC, 90 SZ, and 47 BP	73.9%
Arbabshirani et al. (2013)	Schizophrenia	FNC between independent components extracted by ICA	Different linear and non-linear methods	28 HC and 28 SZ	63–96%
Su et al. (2013)	Schizophrenia	FC between 116 ROIs (AAL atlas)	SVM	32 HC and 32 SZ	82.8%
Yu Y. et al. (2013a)	Schizophrenia	FC between 116 ROIs (AAL atlas)	SVM	38 HC and 32 SZ	80.9
Yu Y. et al. (2013b)	Schizophrenia	FC between 116 ROIs (AAL atlas)	SVM	25 HC, 25 HC sibling, and 24 SZ	62.0%
Guo S. et al. (2014)	Schizophrenia	FC between 90 ROIs (AAL atlas)	SVM	62 HC and 69 SZ	79–82%
Shen et al. (2014)	Schizophrenia	Dynamic ALFF of ROIs	SVM	25 HC and 24 SZ	81.3%
Watanabe et al. (2014)	Schizophrenia	FC between 347 spherical nodes	Lasso, Elastic-net, Graph-net and fused Lasso	67 HC and 54 SZ	59.7–90.8%
Cheng et al. (2015)	Schizophrenia	Graph measures	SVM	29 HC and 19 SZ	80%
Du et al. (2015b)	Schizophrenia, bipolar disorder, and schizoaffective disorder	Network maps from ICA	SVM	24 HC, 24 SZ,24 BP, 24 patients suffering from schizoaffective disorder with manic episodes, and 13 patients suffering from schizoaffective disorder with depressive episodes exclusively	68.75%
Kaufmann et al. (2015)	Schizophrenia	FNC between independent components extracted by ICA	LDA	196 HC and 71 SZ	84.4%
Cabral et al. (2016)	Schizophrenia	FC between 90 ROIs (AAL atlas)	SVM	74 HC and 71 SZ	70.5%
Kim et al. (2016)	Schizophrenia	FC between 116 ROIs (AAL atlas)	Deep neural network	50 HC and 50 SZ	86%
Mikolas et al. (2016)	Schizophrenia	Seed-based FC	SVM	63 HC and 63 SZ	73%
Rashid et al. (2016)	Schizophrenia and bipolar disorder	Static FNC, dynamic FNC and combined static and dynamic FNC	SVM	61 HC, 60 SZ, and 38 BP	59.12–88.68%
Cetin et al. (2016)	Schizophrenia	Static FNC and dynamic FNC from fMRI and MEG data	Linear discriminant classifier (LDC), Naïve Bayes classifier (NBC), and SVM	44 HC and 47 SZ	51.65–90.11%
Skåtun et al. (2016)	Schizophrenia	FNC between independent components extracted by ICA	Regularized LDA	348 HC and 182 SZ	69–78.3%
Liu et al. (2017)	Schizophrenia	Voxel-mirrored homotopic connectivity	SVM	31 HC and 48 SZ	94.93%

*In each of Tables 3–6, the studies are sorted by the year of publication. For each study, we include the brain disorders investigated, the used features, the classifier model, the sample size, and the classification accuracy. It should be noted that evaluating or comparing these studies by only emphasizing the classification accuracy is unfair, since some studies worked on the multi-class classification problem and the sample size and features are greatly varied*.

**Table 4:** Summary of functional connectivity based AD/MCI classification studies.

**References**	**Disorders**	**Features**	**Classifier**	**Sample size**	**Accuracy**
Wang et al. (2006)	Alzheimer	FC of two anti-correlated networks	LDA	14 HC and 14 AD	83%
Chen et al. (2011)	Alzheimer and mild cognitive impairment	FC between 116 ROIs (AAL atlas)	LDA	20 HC, 20 AD and 15 MCI	82%
Wee et al. (2011)	Mild cognitive impairment	FC between WM	SVM	17 HC and 10 MCI	88.9%
Yang et al. (2011)	Alzheimer	Network maps extracted by ICA	SVM	Data1: 316 HC and 100 AD; Data2: 236 HC, 410 MCI and 202 AD	99%
Dai Z. et al. (2012)	Alzheimer	ReHo, ALFF and FC	Maximum uncertainty LDA	22 HC and 16 AD	89.47%
Wee et al. (2012b)	Mild cognitive impairment	FC from fMRI and structural connectivity from sMRI	SVM	17 HC and 10 MCI	96.3%
Wee et al. (2012a)	Mild cognitive impairment	FC between multi-frequency band	SVM	25 HC and 12 MCI	86.5%
Jie et al. (2014)	Mild cognitive impairment	Multi-properties of whole brain FC	Multi-kernel SVM	25 HC and 12 MCI	91.9%
Zhu et al. (2014)	Mild cognitive impairment	FC from fMRI and structural connectivity from sMRI	SVM	18 HC and 10 MCI	95%
Challis et al. (2015)	Alzheimer and mild cognitive impairment	FC between 90-116 ROIs (AAL atlas)	Bayesian Gaussian process logistic regression	39 HC, 27 AD and 30 MCI	75–95%
Dyrba et al. (2015)	Alzheimer	Graph measures from fMRI, and features from DTI and sMRI	Multi-kernel SVM	25 HC and 28 AD	74~85%
Khazaee et al. (2015)	Alzheimer	Graph measures	SVM	20 HC and 20 AD	100%
Zhang X. et al. (2015)	Mild cognitive impairment	FC between 90 ROIs (AAL atlas)	L2-regularized logistic regression	24 HC and 36 MCI	87.5%
Zhang J. et al. (2015)	Alzheimer and mild cognitive impairment	FC between 90 ROIs (AAL atlas)	SVM and k-nearest neighbor	38 HC, 44 early MCI, 38 late MCI, and 26 AD	65.9~73.2%
Chen X. et al. (2016)	Mild cognitive impairment	Dynamic and static FC	SVM	30 HC and 29 MCI	88.1%
Hu et al. (2016)	Mild cognitive impairment	Graph measures	Multi classifier, such as LDA and SVM	20 HC and 30 MCI	97.17%
Liu et al. (2016)	Alzheimer and mild cognitive impairment	FC between 90 ROIs (AAL atlas)	Multiple kernel boosting	230 HC, 200 AD, and 280 MCI	85.8–94.7%
Rahim et al. (2016)	Alzheimer and mild cognitive impairment	Trans-modal learning from PET to fMRI FC	Logistic regression	77 HC, 36 AD, and 98 MCI	76.8%
Schouten et al. (2016)	Alzheimer	FC of fMRI and features of sMRI and DTI	Elastic net	173 HC and 77 AD	93%
Suk et al. (2016)	Mild cognitive impairment	Dynamic state based FC	Auto-Encoder	31 HC and 31 MCI	72.58%
Wee et al. (2016)	Mild cognitive impairment	Dynamic FC	SVM	30 HC and 29 MCI	79.7%
Chen X. et al. (2017)	Mild cognitive impairment	Dynamic FC between GM and WM	SVM	54 HC and 54 MCI	78.7%
Guo H. et al. (2017a)	Alzheimer	Dynamic graph measures	SVM	28 HC and 38 AD	98.16%
Guo H. et al. (2017b)	Alzheimer	FC between 90 ROIs (AAL atlas)	SVM	28 HC and 38 AD	91.60%
Meszlényi et al. (2017)	Mild cognitive impairment	Whole brain FC	Conventional neural network	24 HC and 25 MCI	71.9%
Onoda et al. (2017)	Alzheimer and mild cognitive impairment	Frequency distribution-based FC	SVM	ADNI: 48HC, 33AD and 46MCI; SHIMANE: HC20, AD26, and MCI19	82.6%
Park et al. (2017)	Alzheimer	DMN FC and cortical thickness	SVM	22 HC and 41 AD	91.7%
Sheng et al. (2017)	Mild cognitive impairment	Whole brain FC between voxels	Discriminant analysis	35 HC and 36 MCI	80%
Yu et al. (2017)	Mild cognitive impairment	FC between 90 ROIs (AAL atlas)	SVM	49 HC and 50 MCI	84.8%
De Vos et al. (2018)	Alzheimer	FC, FC dynamics and dynamic FC state	Elastic net and logistic regression	173 HC and 77 AD	70–78%

**Table 5:** Summary of functional connectivity based ASD classification studies.

**References**	**Disorder**	**Features**	**Classifier**	**Sample size**	**Accuracy**
Anderson et al. (2011)	Autism	Whole brain FC between 7266 ROIs	Statistic based classification score	40 HC and 40 ASD	79%
Murdaugh et al. (2012)	Autism	FC between DMN ROIs	Logistic regression	14 HC and 13 ASD	96.3%
Wang et al. (2012)	Autism	FC between 106 ROIs (AAL atlas)	Logistic regression	29 HC and 29 ASD	82.8%
Deshpande et al. (2013)	Autism	FC from fMRI and FA from DTI	SVM	15 HC and 15 ASD	95.9%
Nielsen et al. (2013)	Autism	Whole brain FC between 7266 ROIs	Statistic based classification score	517 HC and 447 ASD	60%
Uddin et al. (2013)	Autism	Independent components extracted by ICA	Logistic regression	20 HC and 20 ASD	78~83%
Zhou et al. (2014)	Autism	Graph measures	SVM and Bayesian network	153 HC and 127 ASD	70%
Chen et al. (2015)	Autism	FC between 220 ROIs from the meta-analysis of functional imaging studies	Random forest	126 HC and 126 ASD	90.8%
Iidaka (2015)	Autism	FC between 90 ROIs (AAL atlas)	Probabilistic neural network	328 HC and 312 ASD	90%
Plitt et al. (2015)	Autism	Whole brain FC from different atlas	SVM	59 HC AND 59 ASD	76.6%
Chen H. et al. (2016)	Autism	Frequency distribution-based FC	SVM	128 HC and 112 ASD	79.2%
Abraham et al. (2017)	Autism	Whole brain FC from different atlas	SVM	468 HC and 403 ASD	68%
Jahedi et al. (2017)	Autism	FC between 220 ROIs from the meta-analysis of functional imaging studies	Conditional random forest	126 HC and 126 ASD	71%
Ktena et al. (2017)	Autism	Graph measures	Convolutional neural network	468 HC and 403 ASD	80%
Sadeghi et al. (2017)	Autism	Local and global functional network properties	SVM	31 HC and 28 ASD	92%
Bernas et al. (2018)	Autism	Time of in-phase coherence between independent component extracted from ICA	LDA and SVM	Data1:18 HC and 12 ASD; Data2: 12 HC and 12 ASD	86.7%
Heinsfeld et al. (2018)	Autism	FC between 200 ROIs (CC200 ROI atlas)	Auto-Encoder	530 HC and 505 ASD	70%

**Table 6:** Summary of functional connectivity-based ADHD classification studies.

**References**	**Disorder**	**Features**	**Classifier**	**Sample size**	**Accuracy**
Zhu et al. (2008)	Attention deficit hyperactivity disorder	ReHo	PCA-FDA	12 HC and 12 ADHD	85%
Bohland et al. (2012)	Attention deficit hyperactivity disorder	FC from fMRI and features from sMRI and non-image features	SVM	168 subjects	74%
Colby et al. (2012)	Attention deficit hyperactivity disorder	FC, graph measures, ReHo from fMRI and features from sMRI	SVM	491 HC, 111 ADHDI, 163 ADHDC, and 11 ADHDH	55%
Dai D. et al. (2012)	Attention deficit hyperactivity disorder	FC, ReHo from fMRI and cortical thickness, gray matter from sMRI	SVM	491 HC and 285 ADHD	67.8%
Dey et al. (2012)	Attention deficit hyperactivity disorder	FC between ROIs detected by proposed algorithm	PCA-LDA	307 HC and 180 ADHD	65.6%
Sato et al. (2012)	Attention deficit hyperactivity disorder	ALFF, ReHo and FC	SVM	546 HC and 383 ADHD	67%
Fair et al. (2013)	Attention deficit hyperactivity disorder	FC between 160 ROIs (Dosenbach atlas)	SVM	455 HC and 193 ADHD	69.2%
Wang et al. (2013)	Attention deficit hyperactivity disorder	ReHo	SVM	23 HC and 23 ADHD	80%
Anderson A. et al. (2014)	Attention deficit hyperactivity disorder	Graph measures from fMRI and features from sMRI	Decision tree	472 HC and 476 ADHD	66.8%
Dey et al. (2014)	Attention deficit hyperactivity disorder	Graph distance measures	SVM	307 HC and 180 ADHD	73.55%
Dos Santos Siqueira et al. (2014)	Attention deficit hyperactivity disorder	Whole brain FC between 400 ROIs	SVM	340 HC and 269 ADHD	77%
Deshpande et al. (2015)	Attention deficit hyperactivity disorder	Directional and non-directional whole brain FC	Artificial neural network	744 HC, 173 ADHDI and 260 ADHDC	90%
Du J. et al., 2016	Attention deficit hyperactivity disorder	FC between PCA selected regions	SVM	98 HC and 118 ADHD	94.9%
Park et al. (2016)	Attention deficit hyperactivity disorder	Whole brain FC between 384 ROIs during task	SVM	13 ADHDI and 21 ADHDC	91.2%
Qureshi et al. (2017b)	Attention deficit hyperactivity disorder	FC from fMRI and features from sMRI	SVM	67 HC, 67 ADHDI and 67 ADHDC	92.9%
Riaz et al. (2017)	Attention deficit hyperactivity disorder	Whole brain FC between ROIs determined using the Affinity Propagation clustering algorithm and non-image data	SVM	NI: 23 HC and 25 ADHD; KKI: 61 HC and 22 ADHD; Peking: 61 HC and 24 ADHD; NYU: 98 HC and 118 ADHD	86.7%

### Schizophrenia and bipolar disorder

Schizophrenia is a severe chronic brain disorder whose symptoms can include delusions, disorganized thinking, hallucinations and social withdrawal (Endicott and Spitzer, 1978; Kay et al., 1987; Calhoun et al., 2008; Fu et al., 2017). Although schizophrenia only affects about 1% of the population worldwide (Bhugra, 2005; Van Os et al., 2010), the symptoms can be very disabling. The symptoms of schizophrenia are categorized into three types: positive, negative and cognitive, and these symptoms usually start in young adulthood and last a long time (American Psychiatric Association, 2013). Bipolar disorder is a mood disorder marked by alternating episodes of mania and depression. Bipolar disorder includes four basic subtypes and all of them involve clear changes in mood, energy, and activity levels (https://www.nimh.nih.gov/health/topics/bipolar-disorder/index.shtml). The root causes of bipolar disorder are not clearly understood, although it is known that both environmental and genetic factors are involved. There is no standard clinical test for either schizophrenia or bipolar disorder. Therefore, it is important to investigate the possibility of using neuroimaging data in the automatic diagnosis of these two brain disorders.

Many studies have focused on distinguishing SZ and HC based on the fMRI functional connectivity. ICA based spatial map is one of the most popular used functional features in the classification (Demirci et al., 2008; Arribas et al., 2010; Castro et al., 2011; Du et al., 2012). For example, Du et al. used ICA to extract individual spatial maps as the initial features and then combined a two-level feature identification scheme with kernel principal component analysis (KPCA) and Fisher's linear discriminant analysis (FLD) in the classification of SZ (Du et al., 2012). By using a majority vote methods that use multiple features, they achieved a classification accuracy of 98% in the auditory oddball task and 93% in the resting-state. The connectivity between identified networks (i.e., FNC) is another important feature for the classification (Anderson and Cohen, 2013; Arbabshirani et al., 2013; Kaufmann et al., 2015). Functional connectivity between ROIs defined by different atlases (i.e., ROI-based) is also commonly used to classify SZs and HCs (Venkataraman et al., 2012; Su et al., 2013; Yu Y. et al., 2013a,b; Watanabe et al., 2014; Kim et al., 2016). Automated anatomical labeling (AAL) atlas is the most popular atlas using in the classification, although some other atlases are also used. Besides these straightforward connectivity features (component spatial maps and functional connectivity), high-level network organization has also been considered as important biomarkers. Bassett et al. (2012) used the size of connected components in graphs build from functional connectivity among time-courses for 90 AAL regions as the input features of SVM and achieved up to 75% classification accuracy and 85% sensitivity. Studies also combined functional connectivity with other features from other modalities to distinguish SZ and HC. Yang et al. proposed a hybrid machine learning method to classify SZs and HCs, using features from fMRI and single nucleotide polymorphism (SNP) data (Yang et al., 2010). They combined three models (SNPs, voxels in the fMRI map contributing to classification and network maps from ICA) into a single module using a majority voting approach to make a final decision. Through a leave-one-out cross-validation, they demonstrated that this framework can provide higher classification accuracy (Combined: 87%; SNP: 74%, voxel: 83%, ICA: 83%). In the 24th Machine Learning for Signal Processing competition (MLSP) (Silva et al., 2014), participants were asked to automatically differentiate 69 schizophrenia patients from 75 healthy controls using multimodal features, including FNC features from fMRI data and component loadings using ICA from structural MRI data. Performance was estimated using the area under the receiver operating characteristic curve (AUC). No entry was able to attain an overall AUC of 0.9 or higher, and the median AUC is near 0.75 across all 2087 entries. The winning team got an overall AUC of 0.89 by means of a Gaussian process (GP) classifier with prior distribution scaled by a probit transformation. Temporal dynamics in the functional connectivity are widely observed in numerous neuroimaging studies and are suggested to be neural origin. Cetin et al. (2016) used static FNC and dynamic FNC obtained from fMRI and MEG data to differentiate schizophrenia patients from healthy controls. They used a leave-one-out cross validation method to examine the classification accuracy. Their results showed that using the combined fMRI and MEG features from FNC improved the classification performance (in which the highest accuracy is 85.71%) compared to using fMRI and MEG FNC features separately (in which the highest accuracy is 75.82%), and using the combined fMRI and MEG features from dynamic FNC improved more (in which the highest accuracy is 90.11%). Increasing studies have demonstrated the benefits of using deep learning in the classification during recent years. Kim et al. (2016) used a L1-norm regularization for feature selection and a deep neural network (DNN) with multiple hidden layers as the classifier. Their results showed that the DNN can obtain about 86% accuracy of two-group classification which is much better than that obtained by SVM.

Functional connectivity-based features for classification of SZ and BP patients at the individual level have been studied as well (Calhoun et al., 2008; Arribas et al., 2010; Rashid et al., 2016). In a previous study (Calhoun et al., 2008), the distance to mean image for each group is constructed using ICA spatial maps of the temporal lobe and the default mode networks. This feature was used in a leave-one-out cross-validation framework, and the approach classified schizophrenia and bipolar patients at the individual level with the accuracy of around 83–95%. A supervised method for automatic classification of healthy controls, patients with bipolar disorder, and patients with schizophrenia using brain imaging data was proposed in Arribas et al. (2010). The spatial maps of independent components were used as the features and a dimension reduction stage comprising two steps is performed (1. *t*-test; 2. singular value decomposition). The reduced features were then used as input of a probabilistic Bayesian classifiers classifier. The experimental results showed that the average three-way correct classification rate (CCR) is in the range of 70–72%, demonstrating their proposed method to be a reliable framework on classification analyses of both schizophrenia and bipolar disorder patients. More recently, time-varying patterns in the functional connectivity have been used to distinguish SZ from BP patients. Rashid et al. proposed a framework for classification of schizophrenia, bipolar and healthy subjects based on their static and dynamic FNC (Rashid et al., 2016). The classification performance between static and dynamic connectivity features was compared through a cross-validation framework. The overall results showed that dynamic FNC (with the classification accuracy 84.28%) significantly outperforms static FNC (with the classification accuracy 59.12%) in terms of predictive accuracy, suggesting that dynamic patterns in functional connectivity might provide distinct and more information over the SFC.

**Figure 3:**
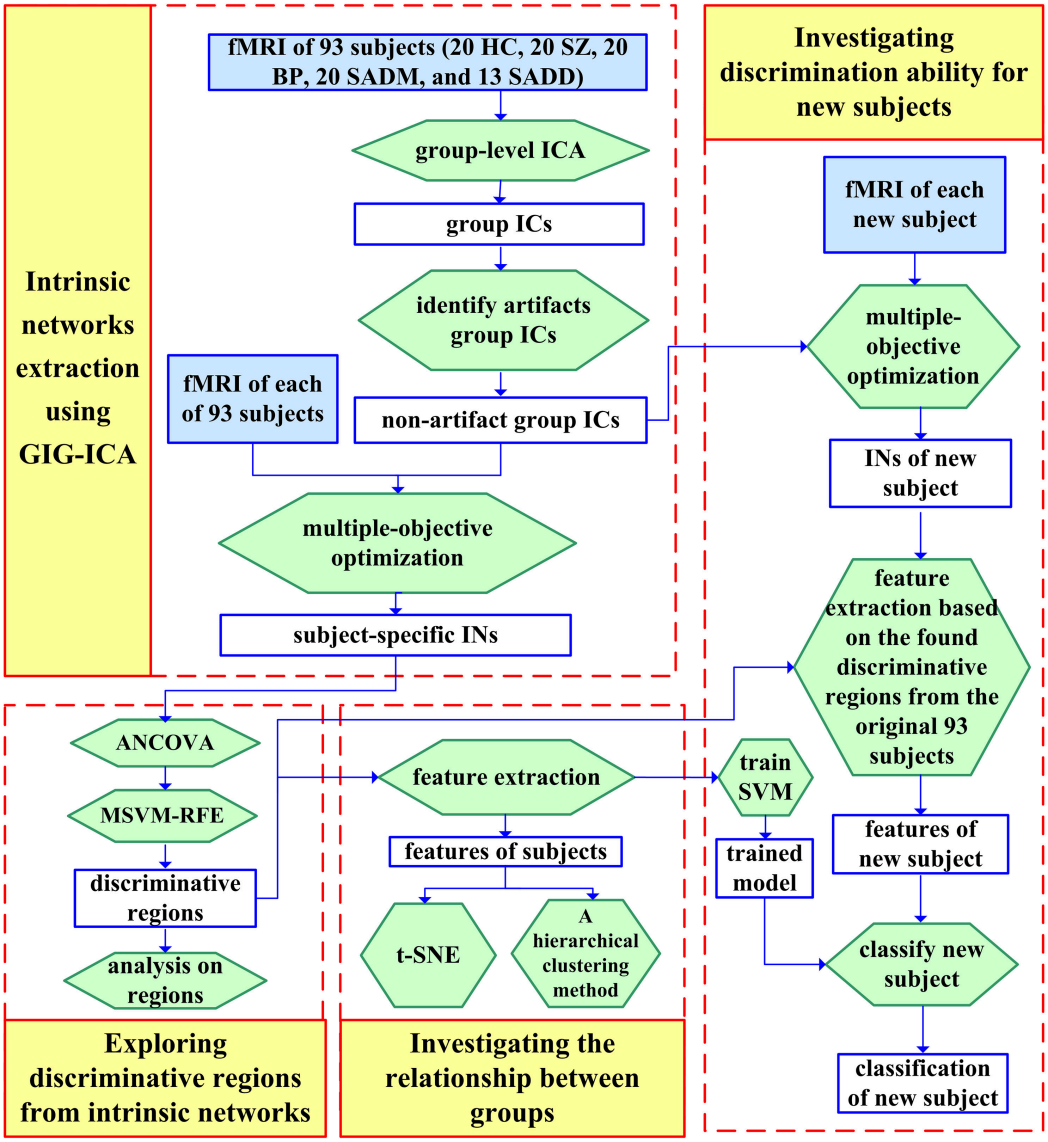
Flowchart of one study (Du et al., 2015b) that includes classifying HCs, SZ patients, BPP patients, SADM patients, and SADD patients. The spatial network maps of the training set computed from GIG-ICA were used as the features in a multiclass (five-class) SVM classifier, that yielded 68.75% classification accuracy for the new coming subjects. The figure is reused with permission from Du et al. (2015b).

**Figure 4:**
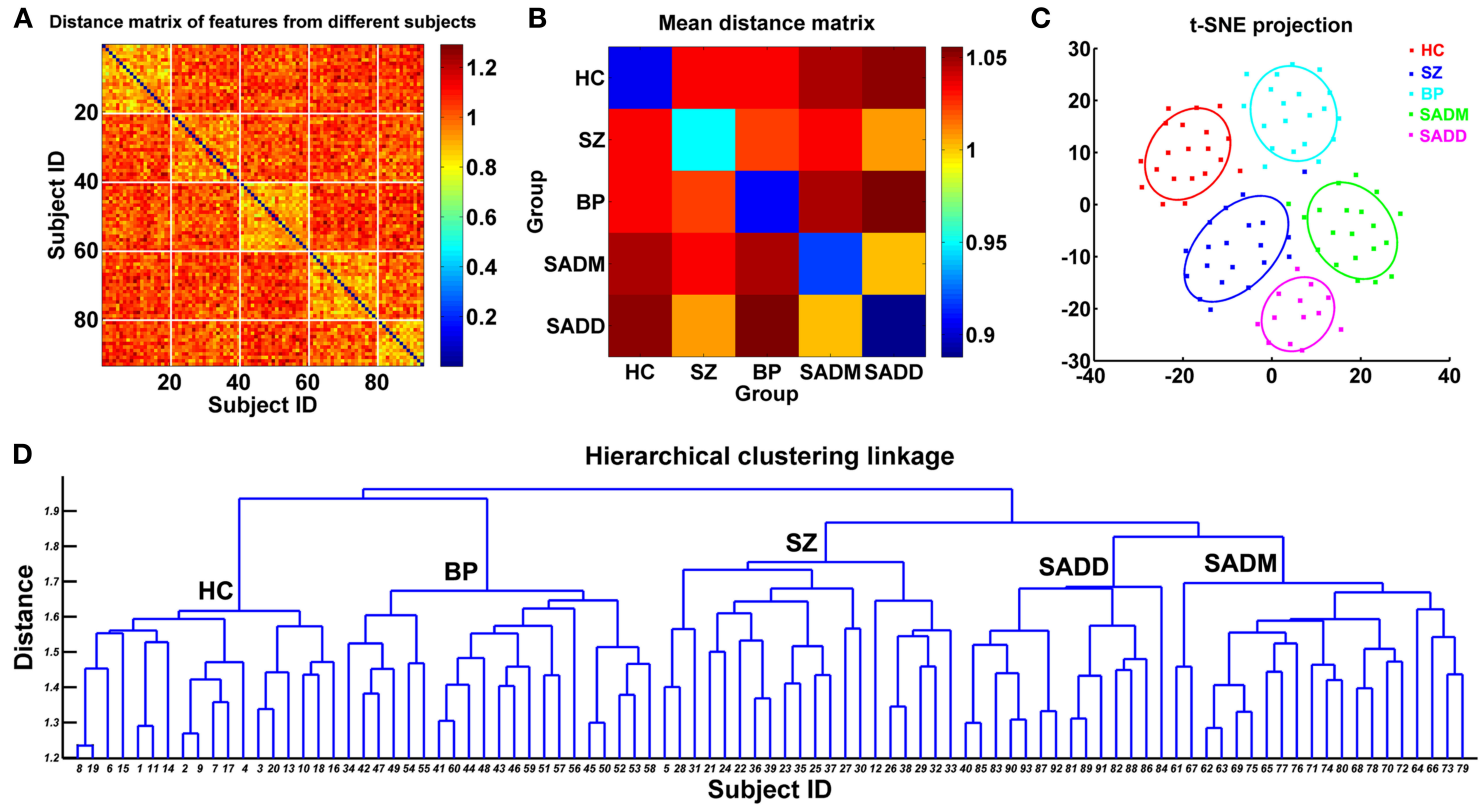
Relationship between those original subjects evaluated using network measures in the study of Du et al. (2015b). **(A)** Distance matrix computed using the feature vectors of 93 subjects. The x-axis and y-axis denote subject ID. Subjects with ID 1–20 are HCs, subjects with ID 21–40 are SZ patients, subjects with ID 41–60 are BP patients, subjects with ID 61–80 are SADM patients, and subjects with ID 81–93 are SADD patients. **(B)** The mean distance matrix obtained by averaging the values in each inter-group and intra-group related sub-block of the distance matrix. **(C)** The projection results of 93 subjects using t-distributed stochastic neighbor embedding (t-SNE) method. Each point denotes one subject, and different colors denote different groups. Each ellipse reflects mean (center) and standard deviation for one group. **(D)** The linkage results from the hierarchical clustering method. The x-axis denotes the subject ID, which is as same as that in **(A)**. In **(D)**, “HC” denotes that most of the subjects clustered into the related group are healthy controls. “SZ,” “BP,” “SADM,” and “SADD” have similar meanings. The figure is reused with permission from Du et al. (2015b).

SZ, SAD, and BPP have overlapping clinical symptoms (Cosgrove and Suppes, 2013; Cardno and Owen, 2014; Pearlson et al., 2016), hence it is very difficult to distinguish them in clinical diagnosis. Du et al. has identified markers from subject-specific brain networks using resting-state fMRI data via GIG-ICA, and then classified healthy controls, SZ patients, BPP patients, patients suffering from schizoaffective disorder with manic episodes (SADM) disorders, and patients suffering from schizoaffective disorder with depressive episodes exclusively (SADD) (Du et al., 2015b). Using the training set, the spatial maps of the typical functional networks were used as the features in a multi-class (five-class) SVM classifier and the RFE was employed for feature selection. For each subject of the testing set, subject-specific networks were computed under the guidance of the group-level networks obtained from the training set, and then the corresponding features were inputted to the classifier trained using the original samples. Results showed that the discriminative regions mainly included frontal, parietal, precuneus, cingulate, supplementary motor, cerebellar, insula and supramarginal cortices, and these regions can provide 68.75% classification accuracy for the new coming subjects (i.e., the independent testing set). Based on measures from functional networks, hierarchical clustering and projection approaches were performed to further investigate the relationship among those groups. Interestingly, the linkage result from the hierarchical clustering showed that using network measures, SADM group and SADD group were closest to each other; SAD group was more similar to SZ group compared to other groups; and BP group was closer to HC group than other patients groups. These results provide an interesting view on the relationship among these symptom-related diseases in addition to accurate separation. The framework and results of this study (Du et al., 2015b) are shown in Figures 3, 4, respectively.

### Autism spectrum disorder and attention deficit hyperactivity disorder

ASD is a complex neurodevelopmental disorder characterized by a wide range of symptoms, skills, and levels of disability that affects how a person acts and interacts with others, communicates, and learns (American Psychiatric Association, 2013). This disorder begins early in childhood and lasts throughout one's life. It is estimated that ASD has a prevalence of 1:68 in the United States (Autism and Developmental Disabilities Monitoring Network Surveillance Year 2008 Principal Investigators; Centers for Disease Control Prevention, 2012) and the lifetime costs of treating an American with ASD has exceeded one million dollars (Greenspan, 2015). The exact cause of autism is still unknown and it might be caused by genetic, brain structure and function, developmental and environmental factors (Wing, 1996). Effective treatments and services can moderate the symptoms and improve the lives. However, ASD is a heterogeneous condition which means there is no same profile for the individuals with ASD and their specific symptoms may change with development (Lord et al., 2000). Consequently, the diagnosis and definition of ASD is still a challenging issue. It is common that children are diagnosed with ASD until ages five and six when is too late for effective treatments. ADHD is another commonly found brain disorder affecting children which share overlapping and confusing symptoms with ASD (Anckarsäter et al., 2006; Happé et al., 2006; Rommelse et al., 2010). Children with ADHD may be inattention, hyperactivity or impulsivity that interferes with school and home life. ADHD is more common in boys than in girls and is usually diagnosed during the early school years and last into adulthood. It is estimated that 3–10% of school-aged children are affected by the ADHD (Biederman, 2005; Dey et al., 2014). The cause of ADHD is still unclear and researchers demonstrate that several things, such as heredity, chemical imbalance, brain changes or injury, and poor nutrition might be involved as possible causes. Currently, a diagnosis of ADHD is mainly based on the behavioral symptoms described in DSM (American Psychiatric Association, 2013). However, DSM can be misleading since there is no valid test for ADHD and ADHD has a high rate of comorbidity, which can confuse matters. Due to the difficulty in diagnosis of ASD and ADHD, an increasing number of studies are using neuroimaging data to develop approaches to try to better characterize and predict these brain disorders. In the following, we review studies using functional connectivity features in the classification of ASD and ADHD.

Studies using functional connectivity as features to classify ASD began around 2011. Anderson et al. calculated the functional connectivity from 7266 ROI covering gray matter during the resting-state and then used these as the features in a thresholding leave-one-out classifier (Anderson et al., 2011). The classifier performed at 89% accuracy for the subjects < 20 years age and at 79% for all subjects. In another study, Murdaugh et al. used seed-based functional connectivity (seed: medial prefrontal cortex, posterior cingulate cortex and angular gyrus) as well as whole-brain functional connectivity in a logistic regression classifier for distinguishing ASD from controls and found that both whole-brain and seed-based connectivity patterns can achieve accuracy up to 96.3% (Murdaugh et al., 2012). The Autism Brain Imaging Data Exchange (ABIDE) initiative has aggregated functional and structural brain imaging data collected from laboratories around the world to accelerate the understanding of the neural bases of ASD (http://fcon_1000.projects.nitrc.org/indi/abide/)(Di Martino et al., 2014, 2017). Plitt et al. used 178 age and IQ matched cohorts from ABIDE and calculated the functional connectivity between three different ROI sets. They used RFE for feature selection in both logistic regression and SVM classifier and obtained an overall 76.7% accuracy of classification (Plitt et al., 2015). Functional connectivity is also combined with the features from other modalities in the classification of ASD. Deshpande et al. identified 18 activated regions from an experiment involving physical and intentional causality and calculated causal connectivity weights, functional connectivity from fMRI, and fractional anisotropy obtained from DTI data for each participant (Deshpande et al., 2013). These features were used in a recursive cluster elimination based SVM classifier and finally achieved a maximum classification accuracy of 95.9%. Deep learning classifiers are applied in the classification of ASD during recent years. Iidaka selected more subjects from ABIDE (312 subjects with ASD and 328 control subjects) and the resting-state functional connectivity between 90 ROIs are used as input of the probabilistic neural network (PNN) for classification. PNN obtained classification results of ~90% accuracy (Iidaka, 2015). Chen et al. constructed functional network between signals in different frequency bands using ABIDE dataset and showed that the most of the discriminative features were concentrated on the Slow-4 band (0.027–0.073 Hz) (Chen H. et al., 2016).

There has also been a fair amount of work using functional connectivity to classify ADHD and healthy controls. Zhu et al. (2008) first used ReHo from fMRI in a PCA-based Fisher discriminative analysis (PC-FDA) to build a linear classifier and the results showed a classification accuracy of 85% using a leave-one-out cross-validation. Wang et al. (2013) extracted ReHo from resting-state fMRI signals and used as input of SVM. They selected features according to a cross-validation procedure and showed that the optimized model produced a total accuracy of 80%. Graph-based measures of functional connectivity are becoming important features that distinguish ADHD from healthy controls (Fair et al., 2013; Dey et al., 2014). Fair et al. used node strength based on the functional connectivity network to successfully classify two subtypes of ADHD (Combined (ADHD-C) and Inattentive (ADHD-I)) from healthy controls with accuracy up to 82.7% (Fair et al., 2013). This graphical measure is also able to separate three groups of cohorts with an overall accuracy of 69.2% in the 3-group classification. Existing studies also use functional connectivity measures along with other fMRI features or other modal features to classify ADHD (Colby et al., 2012; Dai D. et al., 2012; Sato et al., 2012; Anderson A. et al., 2014). For example, Colby et al. combined morphological measures from structural MRI and functional features such as functional connectivity and graphical measures from fMRI as the input features of the SVM and used RFE algorithm for the feature selection. They were able to classify the diagnosis of ADHD with 55% accuracy using this SVM-RFE classifier (Colby et al., 2012). Anderson et al. used functional connectivity measures along with many other features such as curvature index, folding index, Gaussian curvature, gray matter volume, mean curvature, surface area, thickness average, and phenotypic data in a multimodal neuroimaging framework and obtained 66.8% accuracy of two-group classification in an ADHD dataset with a large number of subjects (472 healthy controls and 276 ADHD) (Anderson A. et al., 2014).

Studies have shown that ASD and ADHD have both shared and disorder-specific abnormalities in brain function (Christakou et al., 2013; Chantiluke et al., 2014). However, few studies have used functional connectivity features to distinguish ASD and ADHD and it is still a challenging issue whether functional connectivity can be a powerful biomarker for distinguishing these two brain disorders.

### Alzheimer's disease and mild cognitive impairment

MCI is a syndrome which causes greater memory loss than expected by aging (Gauthier et al., 2006). It is reported that about 3–19% of adults older than 65 years suffer MCI. The symptoms of MCI are not as severe as that in AD and thus people with MCI can carry out their normal daily activities (Albert et al., 2011). There are several subtypes of MCI and one subtype called amnestic MCI which is associated with memory loss has a high risk of progression to AD (Gauthier et al., 2006). Research has shown that the brain areas of memory are impaired in both MCI and AD, while the cognitive domains are only impaired in AD (Petersen et al., 1999). Although the rates of progression varied considerably among literature and the progression is not inevitable, amnestic MCI is still considered to be a forerunner of AD. AD is the most common type of dementia causes problems with memory, thinking and behavior (Strittmatter et al., 1993). AD is increasingly prevalent in individuals over the age of 65 and the significance of AD as a public health problem became evident (Glenner, 1990). It is estimated that 60 new case of AD exists in every hour and by 2050, this number will go to double (Alzheimer's Association, 2015). Between 2000 and 2013, the death results from AD increased remarkably 71%, making AD the sixth leading cause of death in the United States (Alzheimer's Association, 2015).

Traditionally, the diagnosis of AD mainly depends on the clinical examinations and the evaluations of individuals' perception and behavior (Arbabshirani et al., 2017). Improving diagnosis of AD and MCI patients might help to identify diseases earlier in the disease's progress, which may be crucial in developing treatments for these disorders. Considering the severe health impact of AD and MCI and their overall effect on caregivers and society, there has been a large numbers of studies using neuroimaging features, especially the functional connectivity in fMRI to diagnose these brain disorders. Wang et al. proposed a discriminative model of AD based on the Pseudo-Fisher Linear Discriminative Analysis (pFLDA) (Wang et al., 2006). They used the correlation/anti-correlation coefficients of two anti-correlated networks in resting brains as the features of the classification model and obtain a CCR of 83%. Challis et al. employed Bayesian Gaussian process logistic regression (GP-LR) models with linear and non-linear covariance functions in the classification of AD and MCI (Challis et al., 2015). By using functional connectivity as features, they achieved 75% accuracy disambiguating healthy controls from individuals with MCI and 97% accuracy disambiguating individuals with MCI and individuals with AD. Not only the functional connectivity itself, but also its extended or related metrics, such as graphic metrics, have been used as features for the diagnosis of AD and MCI. Jie et al. have developed a novel framework to integrate multiple connectivity properties for improving the diagnosis of MCI (Jie et al., 2014). A multi-kernel learning (MKL) technique was adopted and two types of kernels were used to quantify the local and global connectivity properties respectively. 91.9% classification accuracy was achieved by this method, which is much better than that in previous studies using single connectivity properties. Another study combined graphic theoretical approaches with machine learning method to investigate the atypical functional brain network in patients with AD (Khazaee et al., 2015). They performed statistical analysis on connectivity which is measured by correlation coefficient to search altered connectivity patterns in patients and then calculated three graphic metrics, clustering coefficient, local efficiency, and normalized local efficiency based on the connectivity matrix. A SVM classifier was finally used to explore diagnosis ability of these graphic metrics. Their results showed that those graphic metrics can well separate patients with AD and healthy controls with 100% accuracy. Functional connectivity from fMRI is also incorporated with features from other modalities in the diagnosis of AD. Dai et al. proposed a methodological framework using features from multi-modalities to discriminate patients with AD from healthy controls (Dai Z. et al., 2012). The gray matter volume from structural MRI and three functional characteristics from fMRI were used as the features of classifiers. By using leave-one-out cross-validation, this method provided satisfactory classification accuracy of 89.47% with a sensitivity of 87.50% and a specificity of 90.91%. Schouten et al. used measures from structural MRI, diffusion MRI and resting-state fMRI as the input features of elastic net classifier to classify AD (Schouten et al., 2016). They showed the gray matter density achieved the best classification accuracy among all single modal imaging and multimodal combination can significantly improve the classification performance. These findings suggested that different MRI modalities provide complementary information for classifying AD. The human brain is a dynamic system with non-stationary neural activity and rapidly-changing neural interaction. Increasing evidence shows that functional connectivity is not static but varies significantly in time. There already exist studies using dynamic patterns in functional connectivity as features for the classification of dementia and its pre-stages. A MCI study applied a sliding window approach to estimate dynamic functional correlation tensors between white matters and DFC between gray matters and used these as features to classify MCI subjects (Chen X. et al., 2017). They found that the dynamic functional features significantly improved the classification performance, showing that the functional information in gray matter and white matter is complimentary.

Although vast majority of AD or MCI classification studies used traditional classifiers such as SVM and LDA, increasing studies have considered the advantages of deep learning classifiers over the traditional ones and started using deep learning models in the classification of AD and MCI (Suk et al., 2016; Meszlényi et al., 2017). Meszlenyi et al. described a convolutional neural network for functional connectivity classification called connectome-convolutional neural network (CCNN) (Meszlényi et al., 2017). By testing the performance of CCNN model on both simulated datasets and a public MCI dataset, they showed that the developed model is capable of distinguishing subjects of different groups. Their results also demonstrated that the CCNN model can combine different functional connectivity metrics in the classification and such combination results in better performance than other classifiers using single metric only.

## Challenges and difficulties in identifying biomarkers of brain disorders and classification of individual subject

### Lacking gold standards for diagnoses

Analyzing fMRI data for the ultimate goal of identifying biomarkers and diagnosing brain disorders using neuroimage-based measures is promising but challenging, due to the fact that the current diagnostic categorization itself used as prior guidance could be inaccurate and need further refinement (Insel and Cuthbert, 2015). So far, there is no gold standard for the complex diagnosis. The diagnosis is determined solely by observable symptoms, and the interview and history are the main factors that influence the diagnosis. For example, in clinical diagnosis, it can be difficult to distinguish SZ, BP, and SAD that show overlapping clinical symptoms (Cosgrove and Suppes, 2013; Malaspina et al., 2013; Cardno and Owen, 2014). SZ is a psychotic disorder characterized by altered perception, loss of motivation and judgment, and impairment in social cognition. BP is a mood disorder marked by alternating episodes of mania and depression. SAD is diagnosed when the symptom criteria for SZ are met and during the same continuous period there are major depressive, manic or mixed episodes. In fact, there are also overlapping symptoms such as social withdrawal and communication impairment between ASD and SZ spectrum disorders (Fitzgerald, 2013; Chisholm et al., 2015). ASD, a neurodevelopmental disorder, is characterized by a spectrum of abnormal behaviors including persistent deficits in social communication and interaction across multiple contexts. ADHD is marked by an ongoing pattern of inattention and/or hyperactivity-impulsivity that interferes with functioning or development. Research work also shows a high rate of overlapping symptoms between ASD and ADHD (Taurines et al., 2012). Therefore, the similarities in symptoms between these brain disorders give rise to difficulties in clinical diagnosis.

Most existing fMRI studies (Calhoun et al., 2009a; Koike et al., 2013; Du et al., 2017c), which applied statistical analyses to investigate differences among multiple groups or performed supervised learning approaches to explore biomarkers for effective individual diagnosis and treatment, rely on the diagnostic labeling. The assumptions in those studies are (1) diagnostic groups are distinct from each other and (2) individuals are homogeneous within each predefined group. However, in practice patients could be incorrectly diagnosed due to the overlapping or similar symptoms of diseases, causing that subjects assigned into the same group may show biologically inconsistent alterations. Therefore, the possible bias in the diagnosis labeling will result in inaccurate biomarkers and consequently affect the discriminative power of the classifier constructed based on the provided labels.

There is a great need for the development of disease categories built on biological data and supported by objective and quantitative validation, i.e., the approach recently emphasized by the RDoC initiative (www.nimh.nih.gov/rdoc) (Insel et al., 2010; Cuthbert and Insel, 2013). Due to imperfections of the current disease nosology (especially for psychiatric disorders), how to identify markers/features from a large amount of possibly relevant measures (e.g., high-dimensional neuroimaging data) and then rebuild or refine the nosology based on the neuroimaging-features is a big challenge. One way forward is to consider identifying markers and rebuilding a nosology of disorders (or classifying individual subjects) as one combined problem. The most important and difficult issue is how to propose a “mathematical, precise resolution of what constitutes ‘sufficiently similar' patients” (Djulbegovic and Paul, 2011; Marquand et al., 2016).

### Difficulties in identifying accurate pathological features as biomarkers from high dimensional measures

Given that there are generally more features than samples, it is advantageous to reduce the number of possible measures to focus on a subset of particular interest. As discussed in section Feature Selection Strategies, most relevant work has extracted features in the context of group labeling (e.g., SZ or HC). Even if feature selection is performed using a supervised method, the resulting features are not necessarily able to show a clustering property within each group as expected, since there are usually abundant unrelated and redundant measures considered. In the event the diagnosis is inaccurate, selection of features so that they can show clustering (or similar) patterns within the same group and distinct patterns between different groups is more difficult. Without using group labeling, Clementz et al. (2016) constructed biotypes using a panel of cognitive and electro physiological features that were selected according to known relevance to psychosis and brain function. Promisingly, biotypes showed more reasonable neurobiological heterogeneity and coherent subgroups in psychosis than diagnosis-based category (Clementz et al., 2015; Meda et al., 2016). However, the selected features depended on subjective empirical knowledge and were not automatically extracted from available data. In contrast, some research work (Gates et al., 2014; Geisler et al., 2015; Sun et al., 2015) used all available features and did not further refine features according to prior knowledge. Such selected features working well for one dataset may not converge to a consistent grouping for a different dataset. More advanced methods which can automatically select features that have a good differentiating ability under the condition of no or less guidance of diagnosis labeling are still under way. Semi-supervised feature selection methods (Sheikhpour et al., 2017), which allow using both labeled and unlabeled samples to discover the feature relevance, may be promising and beneficial.

### Challenges in validating biomarkers and classification

Once the biomarkers and biologically-derived classification are obtained, validating the biomarkers and categories (or classification) is another important issue. Most related studies have classified independent subjects based on the identified biomarkers and a well-trained model, and then compared the classification outputs with the diagnosis labels. However, researchers should be aware that the diagnosis labels used as ground-truth could be inaccurate. Some work (Geisler et al., 2015; Clementz et al., 2016) evaluated derived categories using external independent measures or other features that were highly correlated with the used features of the same dataset to see if subjects in one group showed greater similarity in terms of those additional metrics. However, this kind of validation is circular to some extent. A more reasonable technique is to assess biomarker and cluster (or classification) reproducibility by adding additional independent subjects' data or re-sampling of the original data, since a rational classification of brain disorders should be able to map onto pathophysiology using different datasets.

### Other issues that should be considered

There are also other issues which deserve consideration in future clinical applications. In most neuroimaging-based studies focusing on the classification/prediction problem, accuracy, sensitivity, and specificity were used to evaluate the distinguishing ability of the biomarkers identified and the model built. Unlike the screening test (Grimes and Schulz, 2002) that is to detect potential disorders or diseases in people who do not have any symptoms of disease, these assessing metrics (accuracy, sensitivity, and specificity) cannot provide a realistic measure of the positive (probability of having the disease given a positive test) and negative (probability of not having the disorder given a negative test) predictive value (Castellanos et al., 2013), since prevalence of different diseases influences positive/negative predictive value.

In addition to accurately classify the categorization of brain disorders, increasing studies focus on prediction of continuous variables such as individual cognitive scores, symptomatic scores and behavioral performance using fMRI data (Meskaldji et al., 2016; Meng et al., 2017; Shen et al., 2017; Yoo et al., 2018). These studies used different brain connectivity features as the inputs and generate predictors of these features for new coming subjects. Linear regression and partial least square (PLS) regression are the most commonly used methods to achieve the goal. PLS, in which the predictor variables are projected to a new space of components with regard to response variables, is particularly useful, since the number of features is usually much larger than the number of observations/subjects. Support vector regression (Dosenbach et al., 2010), a supervised learning algorithm, which considers all features simultaneously and generates a model that assigns different weights to different features, can also be employed. Generally, the correlation between predicted variables and real recorded variables in the testing set is used to evaluate the performance of the model.

It should be noted that brain diseases can also induce spatial changes due to atrophy for example. In the preprocessing step, inter-subject spatial alignment of fMRI data is typically achieved through registering their co-registered structural MRI images to an anatomic template or directly registering fMRI data to an echo planar imaging (EPI) template. However, these registration methods cannot guarantee fully accurate inter-subject functional consistency, although the following spatial smoothing of fMRI data can reduce the inter-subject functional variability to some extent. Therefore, functional connectivity computed between given brain regions may not accurately correspond across subjects, although the adaptive ICA-based methods are likely more robust to this than ROI or voxel based approaches. In the future, advanced normalization methods (Khullar et al., 2011; Jiang et al., 2013; Cetin et al., 2015) based on function information directly from fMRI data can help address this issue.

## Summary

Mapping brain functional connectivity using fMRI data is now a major emphasis of ongoing research, frequently with a goal of identifying biomarkers and classifying different brain disorders. In this paper, we comprehensively reviewed different approaches which make efforts to accurately map the functional connectome. We included both the traditional static connectivity analysis and the more recently applied dynamic connectivity analysis. Connectivity measures that can be potentially taken as features (i.e., biomarkers) for classification and prediction were clearly summarized for each method. Furthermore, we surveyed various feature selection and classifier building strategies in order to provide guidance on how to perform the classification and predication problem in practice. After that, an updated overview on applications of classifying SZ, BP, ASD, ADHD, were shown. Finally, we discussed gaps in the research and areas that particularly deserve improvement.

## Author contributions

YD proposed the framework and wrote the paper. ZF drafted and revised the paper. VC revised the manuscript and gave final approval.

### Conflict of interest statement

The authors declare that the research was conducted in the absence of any commercial or financial relationships that could be construed as a potential conflict of interest.
